# Genome-Wide Identification and Comparative Analysis of the TCP Transcription Factor Family in Three *Glycyrrhiza* Species Reveals *GuTCP16* as a Positive Regulator of Salt Tolerance

**DOI:** 10.3390/ijms27198446

**Published:** 2026-09-22

**Authors:** Jianting Feng, Kaihua Wei, Xiaomin Xu, Jingyi Zhao, Chunmei Xia, Shi Dong

**Affiliations:** 1 School of Basic Medical Sciences, Xinjiang Second Medical College, Karamay 834000, China; jianting2023@stu.shzu.edu.cn (J.F.); m18546026251@163.com (X.X.); 18997880624@163.com (J.Z.); 2 College of Life Sciences, Shihezi University, Shihezi 832003, China; 3 School of Medicine, Shihezi University, Shihezi 832003, China; weikaihua@stu.shzu.edu.cn

**Keywords:** *Glycyrrhiza* (licorice), TCP transcription factor family, genome-wide analysis, salt stress, *GuTCP16*

## Abstract

The TCP transcription factor family is a plant-specific group of regulators with well-documented roles in mediating abiotic stress responses. Licorice (*Glycyrrhiza*) is a high-value medicinal crop of global economic significance, predominantly cultivated in arid and semi-arid environments where saline soils constrain seed germination, impair seedling development, and diminish both rhizome biomass and the accumulation of pharmacologically active metabolites. Despite the agronomic importance of this genus, a systematic genome-wide survey of the TCP family and its contribution to salt tolerance in *Glycyrrhiza* has not been reported. Here, we identified members of the TCP gene family at the whole-genome level in three representative species—*G. uralensis*, *G. inflata*, and *G. glabra*—and performed integrated bioinformatic characterization encompassing phylogenetic classification, chromosomal distribution, conserved motif architecture, physicochemical parameters, cis-regulatory element composition, and synteny analysis. Transcriptomic profiling revealed that *Glycyrrhiza* TCP genes display pronounced tissue-specific expression and undergo dynamic transcriptional reprogramming under saline conditions. Among the salt-responsive candidates, *GuTCP16* emerged as a key regulator; it was confirmed to localize to the nucleus, and its heterologous expression in Arabidopsis enhanced plant performance under salt stress, demonstrating that *GuTCP16* acts as a positive modulator of salt tolerance. Taken together, this work delineates the evolutionary features and functional diversification of the TCP family in *Glycyrrhiza* and identifies candidate genes to support molecular breeding efforts aimed at developing salt-resilient licorice cultivars.

## 1. Introduction

Licorice (*Glycyrrhiza* spp.), a perennial herbaceous legume, is among the most economically important medicinal crops worldwide, with broad applications in the pharmaceutical and industrial sectors [1].Commercial licorice production is concentrated in arid and semi-arid regions, particularly northwestern China and Central Asia [2], where soil salinization has emerged as a dominant abiotic constraint. *Glycyrrhiza* plants are especially sensitive to salinity during early development: high salt levels reduce seed germination, delay seedling emergence [3], and weaken seedling vigor, thereby compromising subsequent vegetative and reproductive growth and ultimately causing substantial losses in both rhizome yield and the accumulation of pharmacologically active constituents, such as glycyrrhizic acid and flavonoids. Deciphering the molecular basis of salt tolerance and breeding salt-tolerant cultivars are therefore critical for the sustainable development of the licorice industry.

Soil salinity is a major environmental threat that constrains plant growth, crop productivity, and species survival on a global scale [4,5], and its severity is increasing as climate change and inappropriate agricultural practices accelerate soil degradation worldwide [6]. Understanding the molecular mechanisms underlying plant salt stress responses has accordingly become a central topic in plant stress biology, with direct relevance to the molecular breeding of salt-tolerant cash crops. Salt stress damages plants through multiple, interconnected layers. First, the decline in soil water potential imposes osmotic stress that restricts water uptake. Second, excessive accumulation of Na^+^ and Cl^−^ causes ion toxicity and disrupts intracellular ion homeostasis [7]; Third, salt stress triggers a burst of reactive oxygen species (ROS), leading to oxidative damage to cellular membranes, proteins, and nucleic acids [8,9]. These primary injuries propagate into widespread physiological and ultrastructural disorders—including electrolyte leakage, ROS overaccumulation [10], and structural disintegration of organelles such as chloroplasts and mitochondria [11,12]—that collectively inhibit growth and can ultimately induce programmed cell death. Notably, the germination and seedling establishment stages represent the most salt-sensitive period in the *Glycyrrhiza* life cycle [13], underscoring the importance of early-stage tolerance for successful licorice production.

The TCP transcription factor family comprises plant-specific regulators [14] named after its three founding members: TEOSINTE BRANCHED1 (TB1) from maize (*Zea mays*) [15], CYCLOIDEA (CYC) from snapdragon (*Antirrhinum majus*) [16], and PROLIFERATING CELL FACTORS 1 and 2 (PCF1/2) from *Arabidopsis thaliana* [17]. All TCP proteins share a highly conserved N-terminal DNA-binding domain of approximately 60 amino acids, termed the TCP domain, which folds into a non-canonical basic helix-loop-helix (bHLH) structure that mediates DNA recognition and binding [18,19]. The remarkable sequence conservation of this domain across the plant kingdom reflects the ancient evolutionary origin and functional importance of the family.

TCP transcription factors participate in a broad spectrum of biological processes, including growth and development, phytohormone signal transduction, and resistance to biotic and abiotic stresses. In the context of abiotic stress, TCP genes harbor diverse stress-related cis-acting elements in their promoters—such as ABA-responsive elements (ABREs) [20] and anaerobic-responsive elements (AREs)—through which they are integrated into stress-responsive regulatory networks [21]. Functional studies in several species have demonstrated that specific TCP members enhance salt and drought tolerance by modulating abscisic acid (ABA) signaling and downstream stress-responsive genes. In Arabidopsis, for example, *AtTCP13* directly targets IAA5 to promote ABA accumulation and thereby improves drought tolerance [22], whereas overexpression of *AtTCP14* enhances seed germination under salt stress through ABA-dependent signaling. In rice, *OsTCP19* positively regulates salt tolerance by coordinating ion homeostasis and ROS scavenging [23]. Together, these findings establish TCP transcription factors as important regulators of plant salt stress responses and promising targets for improving crop salt tolerance.

Mounting studies have demonstrated that TCP transcription factors are widely involved in plant salt stress responses, and their regulatory functions are closely coupled with the three core damage pathways of salt stress. In response to osmotic stress, TCPs participate in ABA-dependent osmotic adjustment by regulating the transcription of genes involved in ABA biosynthesis and signaling, via ABA-responsive elements (ABREs) in their promoter regions. Regarding ion toxicity, TCPs modulate the expression of ion transporters and channels to maintain intracellular Na^+^/K^+^ balance and alleviate ion poisoning. As for oxidative stress, TCPs transcriptionally activate antioxidant enzyme genes and ROS scavenging systems to mitigate oxidative damage to cellular components.

Genome-wide characterization of the TCP family has been reported for a growing number of plant species, including *Arabidopsis thaliana*, rice (*Oryza sativa*), *Medicago truncatula* [24], and tomato (*Solanum lycopersicum*) [25]. By contrast, the TCP family of *Glycyrrhiza* has not been systematically investigated, and the regulatory roles of its members in salt stress responses remain essentially unknown.

In this study, we performed a genome-wide identification and characterization of the TCP gene family in three widely cultivated *Glycyrrhiza* species—*G. uralensis*, *G. inflata*, and *G. glabra*—based on high-quality reference genomes. The identified TCP members were systematically analyzed with respect to their phylogenetic relationships, chromosomal distribution, conserved motifs, gene structures, promoter cis-acting elements, and intra- and interspecific collinearity. We further examined their tissue-specific expression patterns and salt-responsive expression dynamics by combining transcriptome data analysis with qRT-PCR validation. Finally, the core salt-responsive member *GuTCP16* was characterized in terms of subcellular localization, and its function in salt tolerance was verified by heterologous expression in Arabidopsis. This work provides the first comprehensive view of the evolutionary features and functional diversification of the TCP family in *Glycyrrhiza*, establishes *GuTCP16* as a positive regulator of salt tolerance, and offers valuable candidate genes for the molecular breeding of salt-tolerant licorice cultivars.

## 2. Results

### 2.1. Characterization and Physicochemical Properties of TCP Genes in Glycyrrhiza

Using BLAST (v2.210) searches against the whole-genome sequences of three cultivated *Glycyrrhiza* species, we identified a total of 24, 25, and 24 non-redundant TCP genes from *G. inflata* (Gi), *G. uralensis* (Gu), and *G. glabra* (Gg), respectively (Table S1). All candidate proteins were verified to contain the complete conserved TCP domain—a characteristic non-canonical basic helix-loop-helix (bHLH) structure responsible for DNA binding—via InterProScan and SMART database analyses, confirming their classification as bona fide TCP transcription factor family members.

Detailed physicochemical parameters of all identified TCP proteins are summarized in Table S1. TCP proteins across the three *Glycyrrhiza* species vary widely in length, from 141 amino acids (*GuTCP10*) to 517 amino acids (*GgTCP6*), with corresponding molecular weights ranging from 15.12 kDa to 55.10 kDa. Their theoretical pI values distribute between 5.50 and 11.03, indicating the presence of both acidic and basic TCP proteins in *Glycyrrhiza*. Most TCP family members have an instability index above 40 (ranging from 47.00 to 74.00), suggesting generally poor structural stability under in vitro conditions. Additionally, all TCP proteins show negative GRAVY values, confirming they are hydrophilic soluble proteins. Collectively, the diverse physicochemical features of TCP members imply subtle functional differentiation during *Glycyrrhiza* speciation.

The extreme size variation within the family is not randomly distributed but largely reflects subclass-specific architecture: the shortest protein, *GuTCP10* (141 aa), consists almost exclusively of the conserved TCP domain with minimal flanking regions, whereas the longest member, *GgTCP6* (517 aa), carries extended N- and C-terminal regions outside the TCP domain, which in other species have been associated with regulatory versatility and protein–protein interactions. Notably, members of the same phylogenetic clade showed similar length and pI ranges (Table S1).

### 2.2. Chromosomal Localization of the TCP Gene Family

In this study, we conducted systematic nomenclature and chromosomal localization analysis of TCP gene family members from three *Glycyrrhiza* species (Figure 1), based on official genome annotation data and the physical positions of genes on chromosomes. The results showed that TCP genes were unevenly distributed across all 8 chromosomes in each of the three species.

Comparative analysis revealed highly consistent chromosomal distribution patterns between *G. uralensis* (*GuTCP*) and *G. glabra* (*GgTCP*), with most gene members predominantly enriched on chromosomes 1, 6, and 7. This finding indicates that the TCP gene family has maintained high evolutionary conservation during the speciation and divergence of these two species.

In contrast, *G. inflata* (*GiTCP*) exhibited a markedly species-specific distribution pattern. Its TCP genes were mainly concentrated on chromosomes 3, 4, and 7, and a prominent gene cluster was formed on chromosome 4, represented by *GiTCP2*, *GiTCP5*, *GiTCP14*, *GiTCP10*, and *GiTCP9*. This unique spatial distribution and local aggregation of TCP genes in *G. inflata* may be attributed to lineage-specific evolutionary events during its speciation and long-term evolution, such as gene duplication, chromosomal translocation, and genomic segment deletion.

### 2.3. Phylogenetic Analysis of TCP Proteins Integrated with Conserved Motifs, Conserved Domains and Gene Structure Characterization

To comprehensively resolve the evolutionary position of *Glycyrrhiza* TCP proteins, we constructed an unrooted neighbor-joining phylogenetic tree using full-length TCP protein sequences from the three *Glycyrrhiza* species, together with sequences from three reference species: the model plant *Arabidopsis thaliana* and two representative legumes, *Medicago truncatula* and *Glycine max* (Figure 2).

Across all species, TCP proteins clustered into distinct conserved subfamilies. *Glycyrrhiza* TCPs showed significantly closer phylogenetic affinity with homologs from the two legume species than with those from *A. thaliana*, consistent with their taxonomic relationships. Within the *Glycyrrhiza* genus, orthologous TCP proteins from the three species consistently grouped into tight terminal clades, forming clear orthologous pairs or triplets. This pattern confirms that the majority of TCP family members diverged prior to speciation within the genus, and have retained high sequence conservation during evolution.

Furthermore, we performed an integrative analysis of the structural characteristics of TCP proteins across the three *Glycyrrhiza* species in conjunction with the phylogenetic tree (Figure 3A). Consistent with the topological structure presented in Figure 2, the phylogenetic tree on the left panel of Figure 3 also categorizes all TCP members into distinct subgroups.

Conserved motif analysis (Figure 3B) revealed that TCP proteins clustered within the same evolutionary clade generally share highly similar conserved motif compositions. The vast majority of TCP members harbor several core motifs (e.g., Motif 1 and Motif 2), which reflects the high evolutionary conservation of this gene family. Meanwhile, conserved domain analysis (Figure 3C) showed that nearly all proteins contain the canonical “TCP superfamily” or “TCP2 superfamily” domain, providing robust evidence supporting their classification as bona fide TCP family members. The highly consistent motif arrangement and domain architecture within the same clade lay a solid foundation for further exploring the evolutionary-functional relationships of *Glycyrrhiza* TCP genes.

### 2.4. Analysis of Cis-Acting Elements in the Promoter Regions of TCP Genes

Cis-acting elements in promoter regions serve as core regulatory determinants of gene expression patterns. To explore the putative regulatory network of TCP genes in *Glycyrrhiza*, we systematically extracted the 2000 bp sequences upstream of the translation start codon (ATG) of each family member for quantitative and positional analysis. The results showed that most TCP genes exhibited marked differences in both the type and number of cis-elements. This high heterogeneity suggests that the TCP family has undergone extensive functional divergence throughout long-term evolution. Interestingly, members with close phylogenetic relationships generally retained highly similar element composition profiles. This structure-evolution coupling pattern can be largely attributed to the linked inheritance of promoter sequences during gene duplication events (Figure 4A).

Spatial distribution analysis revealed that the promoter regions of *Glycyrrhiza* TCP genes function as sophisticated signal integration hubs. The abundant and diverse cis-acting elements endow these genes with the capacity to sensitively perceive both endogenous and exogenous environmental cues. In terms of phytohormone signaling pathways, abscisic acid (ABA)-responsive elements (ABREs) and methyl jasmonate (MeJA)-responsive elements (CGTCA/TGACG-motifs) are the most predominant, while salicylic acid (SA)-responsive elements (TCA-elements) and auxin-responsive elements (TGA-elements) are also widely distributed across these promoters. Meanwhile, abiotic stress-responsive elements are also densely distributed (Figure 4B). For instance, anaerobic induction elements (AREs), low-temperature responsive elements (LTRs), and drought-induced MYB binding sites (MBSs) are frequently detected in multiple TCP gene sequences. These diverse regulatory elements are intertwined to form a molecular defense network that enables *Glycyrrhiza* to cope with complex environmental stresses.

Quantitative analysis based on the heatmap further uncovered the commonalities and specificities of cis-element distribution across the TCP family. Overall, ABREs and AREs exhibit the widest distribution and highest enrichment level among the vast majority of TCP genes, and these two dominant cis-elements make distinct functional contributions to the salt stress response. ABRE (ABA-responsive element) serves as the core cis-regulatory motif mediating ABA-dependent salt stress signaling. It primarily acts during the osmotic stress phase and the long-term acclimation stage of salt stress, triggering the transcriptional activation of TCP genes in response to ABA signals. In this way, ABRE-associated TCPs participate in stomatal regulation, osmolyte accumulation, and the gradual establishment of stress tolerance. In contrast, ARE (anaerobic-responsive element) is predominantly linked to the response to hypoxic stress and energy metabolism disruption, both of which arise from salt-induced osmotic stress and restricted root respiration. ARE exerts a more pronounced function in the early phase of salt stress and in the adaptation of root tissues to high-salinity environments.

The combined enrichment of ABRE and ARE in TCP gene promoters allows TCP family members to engage in multiple temporal phases and tissue contexts of the salt stress response via independent signaling pathways, collectively forming the sophisticated stress regulatory network in *Glycyrrhiza*. Notably, the promoter regions of certain genes show a dramatic accumulation of stress-responsive elements, indicating strong stress tolerance potential and unique functional specificity.

Taken together, the highly flexible promoter architecture enables TCP genes to comprehensively integrate complex phytohormone networks and multiple abiotic stress signals, thereby exerting irreplaceable regulatory functions in plant growth, development, and environmental adaptation.

### 2.5. Synteny Analysis of the TCP Gene Family

To elucidate the evolutionary dynamics of the TCP gene family within the *Glycyrrhiza* genus, we conducted a systematic genome-wide collinearity analysis across three representative species: *G. inflata*, *G. uralensis*, and *G. glabra*. The intra-genomic collinearity landscapes are illustrated in circular plots (Figure 5A–C), where the outermost track displays genome-wide gene density and the inner track indicate GC content.

Our analysis revealed that segmental duplication events are pervasive across the genomes of all three *Glycyrrhiza* species, and represent the dominant genetic mechanism driving the expansion of the TCP gene family. Specifically, we identified 11, 12, and 11 collinear TCP gene pairs in *G. glabra*, *G. uralensis*, and *G. inflata*, respectively.

To decipher the selection pressure and evolutionary timing of these duplication events, we calculated the non-synonymous substitution rate (Ka), synonymous substitution rate (Ks), and Ka/Ks ratio for all collinear TCP gene pairs (full results listed in Table S5). All collinear pairs had Ka/Ks ratios below 1, with most values lower than 0.5, demonstrating that these duplicated TCP loci have experienced strong purifying selection over evolutionary time. This selection constraint has maintained the sequence conservation of the core TCP domain and preserved the structural and functional integrity of TCP proteins after duplication. We further estimated the occurrence time of these segmental duplication events based on Ks values. The results indicated that most duplication events took place in the relatively recent evolutionary history of the *Glycyrrhiza* genus, supporting that recent segmental duplication acts as a major driver of TCP family expansion in this lineage.

Building on the intra-species collinearity results, we further performed interspecific synteny analysis to investigate the evolutionary relationships among the three *Glycyrrhiza* species (Figure 5D). The results demonstrated extensive and strong syntenic conservation across the three genomes, with a large number of orthologous TCP gene pairs retaining clear orthologous correspondence between species. This cross-species syntenic association not only delineates the speciation and divergence trajectory of the *Glycyrrhiza* genus, but also verifies the high genetic conservation of TCP genes across different *Glycyrrhiza* lineages. Collectively, these findings provide robust genomic evidence for further exploring the functional homology of TCP genes among *Glycyrrhiza* species.

### 2.6. Expression Profiling Analysis of the TCP Gene Family

In this study, we performed systematic clustering analysis based on transcriptome data of *G. inflata*, *G. uralensis*, and *G. glabra* under 2 h and 24 h salt stress treatments, to comprehensively characterize the expression response patterns of the TCP gene family under salt stress. The results showed that the transcription levels of numerous TCP genes changed significantly at the early stage of salt treatment (2 h), exhibiting a rapid stress response feature. With stress duration extended to 24 h (Figure 6A), the overall expression profile of TCP genes underwent dynamic remodeling, showing a sustained adaptive regulatory trend. The salt-induced expression patterns of TCP genes across the three species exhibited both evolutionary conservation and interspecific divergence, suggesting that the salt stress regulatory pathways of TCP family members have undergone varying degrees of functional differentiation during *Glycyrrhiza* speciation.

To clarify the spatial characteristics of TCP genes in exerting biological functions, we further analyzed the tissue-specific expression profiles of all TCP genes in roots, stems, and leaves (Figure 6B). The results revealed that most TCP genes exhibited obvious tissue expression bias: some family members were preferentially enriched in leaves, while others were predominantly highly expressed in roots. This spatial expression heterogeneity reflects that different TCP family members are respectively involved in regulating *Glycyrrhiza* growth and development, organ morphogenesis, and basic physiological metabolism, undertaking delicate and differentiated biological functions.

To verify the accuracy and reliability of the transcriptome sequencing results, we selected 8 representative *G. uralensis* TCP genes for expression validation via quantitative real-time PCR (qRT-PCR). The results showed that the temporal expression trends of the selected genes were highly consistent with the transcriptome analysis results during the 0–24 h salt stress treatment (Figure 6C). Among them, *GuTCP13* and *GuTCP16* showed the most significant response to salt stress, and the expression level of *GuTCP13* was up-regulated by approximately 4.4-fold at 2 h after salt treatment compared with the 0 h control. These validation results further confirm that TCP family genes play a crucial role in environmental stress adaptation and growth regulation of *Glycyrrhiza*.

### 2.7. Subcellular Localization and Salt Stress Tolerance Function Analysis of GuTCP16

To clarify the subcellular localization characteristics of the *GuTCP16* protein, we constructed a *GuTCP16*-GFP fusion expression vector driven by the 35S constitutive promoter, and performed localization analysis via an Agrobacterium-mediated transient transformation system in tobacco leaves (Figure 7A). The results showed that in the control group transformed with the empty 35S: GFP vector, green fluorescent signals were widely distributed in both the nucleus and cytoplasm with no obvious localization specificity. In contrast, the green fluorescence signal of the 35S:*GuTCP16*-GFP fusion protein completely colocalized with the red fluorescence signal of the mCherry nuclear localization marker, and was exclusively detected in the nucleus. This result confirms that *GuTCP16* is a nuclear-localized protein, which is consistent with the fundamental characteristic of transcription factors that exert transcriptional regulatory functions in the nucleus.

To further investigate the biological function of *GuTCP16* in salt stress response, we used wild-type *Arabidopsis thaliana* (WT), *attcp16* loss-of-function mutant, *GuTCP16* complementation line (*GuTCP16*/*attcp16*) and *GuTCP16* overexpression line (*GuTCP16*-OE) as experimental materials, to compare the growth phenotypic differences of plants with different genotypes under salt stress (Figure 7B). Under normal growth conditions, there was no significant difference in growth status among all lines, and no obvious phenotypic differentiation was observed. After salt stress treatment, the *attcp16* mutant showed significantly more severe wilting and higher leaf yellowing ratio than the wild type, presenting a typical salt-sensitive phenotype. The *GuTCP16*-OE overexpression line exhibited stronger stress tolerance, and its overall growth status was significantly better than that of the wild type. In addition, the complementation line could restore the salt-sensitive phenotype of the *attcp16* mutant to the wild-type level. These results indicate that *GuTCP16* plays a positive regulatory role in the plant salt stress response process.

## 3. Discussion

The TCP family is a plant-specific transcription factor family with well-established functions in growth, development, and abiotic stress adaptation [26]. Although genome-wide surveys of TCP genes have been reported for numerous plant species [27], no systematic characterization had been performed in *Glycyrrhiza*, and the involvement of TCP members in the salt stress response of this genus remained unknown. By integrating genome-wide identification across three medicinal *Glycyrrhiza* species (*G. uralensis*, *G. inflata*, and *G. glabra*) with expression profiling and functional validation, the present study provides the first comprehensive picture of the evolution, structure, and stress responsiveness of the *Glycyrrhiza* TCP family, and establishes *GuTCP16* as a positive regulator of salt tolerance.

Phylogenetic reconstruction placed the TCP proteins of the three *Glycyrrhiza* species into well-resolved clades, within which orthologs from the three species consistently grouped together at the branch tips, forming orthologous pairs or three-species orthologous groups. This topology indicates that most TCP members arose before the divergence of the three species and have remained highly conserved since then [28]. Chromosomal mapping further revealed an uneven distribution of TCP genes across the eight chromosomes of each species [29]. The distribution patterns of *G. uralensis* and *G. glabra* were nearly identical, with members concentrated on chromosomes 1, 6, and 7, consistent with the close evolutionary relationship between these two species. In contrast, *G. inflata* displayed a species-specific pattern centered on chromosomes 3, 4, and 7, including a pronounced gene cluster on chromosome 4. This lineage-specific clustering likely reflects local gene duplication, chromosomal translocation, or fragment loss events following speciation [30], highlighting divergent trajectories of genome structural evolution within the genus.

Gene structure and conserved motif analyses reinforced the phylogenetic grouping: members of the same subgroup shared nearly identical motif compositions and domain architectures, whereas clear differences separated the subgroups. Almost all identified proteins retained the canonical TCP domain—a non-canonical bHLH fold that defines the family and is highly conserved across the plant kingdom [26]—supporting their annotation as bona fide TCP transcription factors. The structural coherence within subgroups implies functional conservation among co-clustered homologs, whereas the inter-subgroup divergence in exon–intron organization and motif content points to functional differentiation during the evolution of the family, potentially allowing different members to fulfill distinct regulatory roles in development and stress adaptation. Similar conservation-within-clade and divergence-between-clade patterns have been documented in the TCP families of Arabidopsis, rice, and cotton [31], suggesting that this is a general feature of plant transcription factor family evolution.

Gene duplication is a principal driver of gene family expansion and functional innovation. Intraspecific synteny analysis identified multiple collinear TCP gene pairs in each of the three *Glycyrrhiza* species, the majority of which arose from segmental rather than tandem duplication, indicating that segmental duplication was the predominant mechanism of TCP family expansion in this genus. A similar segmental-duplication-dominated mode has been reported for the WRKY family in *Medicago truncatula*, suggesting that this expansion route is common among plant stress-related transcription factor families. Segmental duplicates often retain ancestral functions, and the resulting redundancy can buffer stress regulatory networks against perturbation; at the same time, duplicated copies may undergo sub- or neofunctionalization, increasing regulatory plasticity. For *Glycyrrhiza* species inhabiting arid and semi-arid regions with widespread soil salinization, segmental-duplication-driven expansion of the TCP family may therefore represent an evolutionary strategy contributing to long-term adaptation to salt and drought stress. Interspecific synteny analysis further revealed extensive collinearity among the three species, corroborating their close genetic relationship [32] and providing a framework for transferring functional annotations from model plants to *Glycyrrhiza* TCP genes.

Promoter cis-acting elements act as molecular switches that determine when and where a gene is transcribed, and they are central to the integration of stress and hormone signals [33]. The promoters of *Glycyrrhiza* TCP genes were enriched in stress- and hormone-responsive elements, among which ABA-responsive elements (ABREs) and anaerobic-responsive elements (AREs) were the most abundant and widely distributed. This enrichment suggests that ABA signaling occupies a central position in the transcriptional regulation of *Glycyrrhiza* TCP genes. ABA is a key phytohormone in the salt stress response, enhancing tolerance through the regulation of stomatal movement, osmolyte accumulation, ion homeostasis, and antioxidant defense [34], and several TCP transcription factors have been shown to act within ABA-dependent pathways by directly regulating downstream stress-responsive genes [35]. Elements responsive to methyl jasmonate, salicylic acid, and auxin, together with low-temperature-responsive elements (LTRs) and drought-inducible MYB-binding sites (MBSs), were also broadly represented, indicating that TCP genes can integrate multiple hormonal and environmental cues and thus function pleiotropically in *Glycyrrhiza* growth and stress adaptation. Comparable cis-element repertoires in the TCP families of other species further imply that the stress responsiveness of TCP genes is evolutionarily conserved in plants.

The spatiotemporal expression profile of a gene provides important clues to its biological function [36]. *Glycyrrhiza* TCP genes displayed clear tissue-preferential expression, with different members predominating in roots, stems, or leaves, indicating that family members have partitioned organ-specific functions. Because roots are the primary site of salt perception and of water and nutrient uptake, root-preferential TCP genes are candidates for direct involvement in salt signal perception, root architecture remodeling, and ion homeostasis under salinity [37]. Leaf-preferential members, in contrast, may contribute mainly to the protection of the photosynthetic machinery, ROS detoxification, and the maintenance of leaf growth, thereby mitigating salt damage to aerial tissues [38].

Salt stress transcriptome analysis further resolved the temporal dynamics of the TCP response. A large fraction of TCP genes changed significantly in expression as early as 2 h after salt exposure, demonstrating that the family responds rapidly to salt signals and participates in the early phase of the stress response. By 24 h, the expression profile had been substantially remodeled, consistent with sustained regulatory roles during long-term acclimation. The response patterns were partly shared and partly divergent among the three species, reflecting both the conservation of salt-responsive mechanisms within the genus and the differentiation of regulatory pathways during adaptation to distinct habitats [39]. qRT-PCR assays of eight representative *GuTCP* genes produced expression trends highly concordant with the transcriptome data, supporting the reliability of the RNA-seq results. Among the genes examined, *GuTCP16* exhibited the strongest and most sustained salt-induced up-regulation, nominating it as a priority candidate for functional dissection.

Subcellular localization assays confirmed that *GuTCP16* is a nuclear protein, consistent with its predicted role as a transcription factor. Functional evidence came from heterologous expression in Arabidopsis: under control conditions, the *attcp16* mutant, *GuTCP16*-overexpression lines, and complementation lines were phenotypically indistinguishable from the wild type, whereas under salt stress the *attcp16* mutant displayed more severe wilting and chlorosis, overexpression lines showed markedly improved growth, and complementation lines restored the wild-type phenotype. Collectively, these phenotypic findings support that *GuTCP16* acts as a positive regulator of the salt stress response. However, the current functional evidence is mainly derived from phenotypic observations; the underlying physiological and biochemical mechanisms, including changes in antioxidant enzyme activities, ROS accumulation levels, osmolyte contents and ionic homeostasis, remain to be further elucidated. Notably, the ability of *GuTCP16* to rescue the Arabidopsis *attcp16* mutant indicates that the salt-regulatory function of TCP16 orthologs is conserved across these distantly related species, in line with reports that TCP overexpression enhances salt tolerance in Arabidopsis and rice by up-regulating downstream stress-responsive genes [23]. Combined with the enrichment of ABRE cis-elements in the *GuTCP16* promoter, the conserved salt tolerance function of TCP homologs across other plant species, and the salt-responsive expression profile observed in this study, we speculate that *GuTCP16* may participate in the salt stress response via an ABA-dependent signaling pathway, and thereby modulate plant ion homeostasis, oxidative damage repair and osmotic adjustment. However, this hypothesis is currently based on bioinformatic prediction and functional inference from homologous genes, and direct experimental evidence supporting the involvement of ABA signaling remains absent. Verification of this specific regulatory pathway will require quantification of endogenous ABA levels and expression analysis of ABA signaling marker genes in future work. Direct testing of this model will require the identification of its downstream targets.

In conclusion, this study provides the first genome-wide characterization of the TCP transcription factor family in three *Glycyrrhiza* species, covering their evolutionary relationships, structural features, duplication history, cis-regulatory architecture, and expression behavior under salt stress, and functionally verifies *GuTCP16* as a positive regulator of salt tolerance. These findings advance our understanding of TCP-mediated stress regulation in *Glycyrrhiza* and supply candidate genes for breeding salt-tolerant licorice. Several directions merit further investigation. First, the downstream targets of *GuTCP16* should be identified through approaches such as yeast one-hybrid screening and ChIP-seq to resolve the regulatory network it controls under salinity. Second, because functional validation was performed in a heterologous system, stable genetic transformation of *Glycyrrhiza* will be essential to confirm the in vivo role of *GuTCP16* and to evaluate its breeding value. Such efforts should ultimately support the development of licorice cultivars combining high salt tolerance with desirable medicinal quality.

## 4. Materials and Methods

### 4.1. Plant Materials and Stress Treatments

Seeds of *G. uralensis* received surface disinfection prior to sowing in pots filled with a 3:1:1 (*v*/*v*/*v*) mixture of nutrient soil, vermiculite and perlite. All seedlings were maintained in a climate-controlled chamber under 25 °C, 16 h light/8 h dark photoperiod and 70% relative humidity, and were irrigated with half-strength Hoagland (Solarbio, Beijing, China) solution weekly. Uniform seedlings at the three-leaf stage were selected and exposed to NaCl (200 mmol/L) salt stress. Fully expanded functional leaves from the upper part of seedlings were sampled at preset time points after treatment, immediately snap-frozen in liquid nitrogen, and stored at −80 °C for subsequent RNA extraction and quantitative verification.

*Arabidopsis thaliana* ecotype Columbia-0 (wild type) was used as the wild-type control. The *attcp16* homozygous T-DNA insertion knockout line (SALK_061068), with a Col-0 genetic background, was acquired from the Arabidopsis Biological Resource Center (ABRC). The T-DNA fragment is inserted into the coding region of *AtTCP16* (locus: AT3G45150), generating a complete loss-of-function null allele. Homozygosity of the mutant was verified by PCR genotyping before experimental use. Seed sterilization, vernalization and cultivation were performed following widely used standard protocols. Briefly, surface-sterilized seeds were vernalized at 4 °C for 3 days, sown on 1/2 MS solid medium, and cultured in a growth chamber at 22 °C with a 16 h light/8 h dark cycle. Seven-day-old seedlings were transplanted into nutrient soil for further growth. For salt tolerance phenotyping, 3-week-old plants of each genotype were treated with NaCl solution, and growth status was observed and recorded.

*Nicotiana benthamiana* plants were grown under the same conditions as *Arabidopsis*, and 4-week-old seedlings were used for transient transformation in subcellular localization assay.

### 4.2. Genome-Wide Identification of TCP Family Members

Whole-genome sequence datasets of the three *Glycyrrhiza* species applied in this research were provided by South China Agricultural University [40]. Full-length TCP protein sequences of *Arabidopsis thaliana* were downloaded from the TAIR database and served as query sequences. TBtools (v2.210) with an embedded BLASTP algorithm was used to perform sequence alignment against local whole-genome protein databases of the three *Glycyrrhiza* species, with a cutoff E-value of 1.0 × 10^−10^ to reduce false-positive matches. Candidates sharing at least 60% sequence identity with *Arabidopsis* TCP proteins were retained for further validation.

Conserved domains of candidate proteins were predicted and verified via the InterProScan and SMART databases. Sequences without the complete typical TCP conserved domain were excluded, and the remaining sequences were confirmed as final TCP family members. Additionally, physicochemical parameters of each TCP protein, including molecular weight (MW) and isoelectric point (pI), were calculated using the pI/Mw tool on the ExPASy server 3.0 (SIB Swiss Institute of Bioinformatics, https://www.expasy.org/), accessed in 2026.

### 4.3. Phylogenetic Reconstruction

To clarify the evolutionary status of *Glycyrrhiza* TCP proteins across plant lineages, phylogenetic analysis was performed using MEGA 11 software. In addition to the three *Glycyrrhiza* species, TCP protein sequences from three representative reference species were included: *Arabidopsis thaliana* (retrieved from the TAIR database), and two legume species *Medicago truncatula* and *Glycine max* (both retrieved from the Phytozome database). Multiple sequence alignment was conducted with the ClustalW algorithm, and a neighbor-joining (NJ) phylogenetic tree was constructed with 2000 bootstrap replicates and genetic distance calculated using the Poisson correction model. The resulting phylogenetic tree was visualized and optimized on the iTOL online platform.

### 4.4. Chromosomal Distribution Analysis

Chromosomal location information of all TCP genes was retrieved from the corresponding genome annotation datasets of the three *Glycyrrhiza* species. The spatial distribution pattern of TCP genes across chromosomes was visualized using TBtools software, with chromosome length and gene position marked correspondingly.

### 4.5. Analysis of Gene Structure, Conserved Motifs and Functional Domains

The MEME online platform was employed to predict conserved motifs in TCP protein sequences, with the maximum number of motifs set to 10. Conserved functional domains were identified using the NCBI CD-Search online tool. Exon-intron structure information of TCP genes was extracted from genome annotation files. The phylogenetic tree, conserved motifs, functional domains and gene structures were integrated and visualized via TBtools software.

### 4.6. Cis-Acting Element Prediction in Promoter Regions

The 2000 bp genomic sequences upstream of the translation initiation codon (ATG) of each TCP gene were extracted as promoter regions. Cis-regulatory elements in these promoter sequences were predicted and annotated using the PlantCARE database. The type, quantity and distribution of cis-elements were counted and summarized, and the results were visualized with TBtools software.

### 4.7. Synteny Analysis

Synteny analysis of TCP genes from the three *Glycyrrhiza* species was performed using the built-in One Step MCScanX function of TBtools software. Both intra-species collinearity within each species and inter-species synteny across the three species were analyzed to identify gene duplication events and orthologous gene pairs. Syntenic relationships were displayed by Circos plots, and the core driving force for TCP gene family expansion was further explored.

### 4.8. Expression Profile Analysis of TCP Genes

To characterize the tissue expression specificity and salt stress response pattern of TCP genes, publicly available transcriptome data of the three *Glycyrrhiza* species covering multiple tissues and salt stress time points were used for expression profile analysis. FPKM values of TCP genes were extracted and normalized via log_2_ transformation (log_2_(FPKM + 1)), and expression heatmaps were generated using TBtools.

### 4.9. Quantitative Real-Time PCR Validation

Total RNA was isolated from licorice leaf samples using a plant total RNA extraction kit (TIANGEN, Beijing, China)). After quality and integrity detection, total RNA was reverse-transcribed into cDNA. The *UBQ* gene of *Glycyrrhiza* was selected as the internal reference, and gene-specific primers were designed for qRT-PCR validation (primer sequences are listed in Supplementary Table S2). qRT-PCR reactions were performed with SYBR Green qPCR mix (TIANGEN, Beijing, China) according to the manufacturer’s instructions. Each sample was set with three biological replicates and three technical replicates. The relative expression level of each gene was calculated using the 2^−ΔΔCt^ method.

### 4.10. Subcellular Localization Assay

The full-length coding sequence (CDS) of *GuTCP16* (without stop codon) was PCR-amplified using *G. uralensis* cDNA as template. The target fragment was inserted into the *pCAMBIA1300-GFP* vector via homologous recombination to construct the 35S:*GuTCP16*-GFP fusion expression vector driven by the 35S constitutive promoter, with the empty 35S: GFP vector serving as negative control.

The sequence-verified recombinant vector was transformed into *Agrobacterium tumefaciens* strain GV3101 (WeidiBio, Shanghai, China). Positive clones were confirmed by PCR and then infiltrated into leaves of 4-week-old *N. benthamiana*. After 48 h of dark incubation, green fluorescent signals in leaf epidermal cells were observed under a laser confocal microscope. The 35S-mCherry-NLS vector was used as a nuclear marker for co-localization confirmation.

### 4.11. Vector Construction and Arabidopsis Transformation

The full-length CDS of *GuTCP16* was inserted into the *pCAMBIA1300* (Maokang Bio, Shanghai, China) overexpression vector to generate the 35S:*GuTCP16* overexpression construct. The recombinant plasmid was transformed into *Agrobacterium tumefaciens* strain GV3101, and then introduced into wild-type Col-0 and *attcp16* mutant plants respectively via the Agrobacterium-mediated floral dip method, to produce overexpression lines and complementation lines. Transgenic plants were screened by hygromycin resistance and verified by PCR. T_3_ homozygous lines were used for subsequent salt stress phenotyping assays.

### 4.12. Statistical Analysis

Statistical analysis and graph plotting were conducted using GraphPad Prism (v10.1.2). To quantify leaf yellowing under salt stress, the total leaf area and yellowed leaf area of each plant were measured using ImageJ (1.54f, National Institutes of Health, Bethesda, MD, USA). The leaf yellowing ratio was calculated as the percentage of yellowed leaf area relative to the total leaf area, with at least 6 individual plants per genotype included for quantification. Student’s *t*-test was applied to analyze differences between two groups, and Duncan’s multiple range test was used to assess differences among multiple groups. Significance levels were defined as follows: *p* > 0.05 (no significant difference), *p* < 0.05 (significant difference), and *p* < 0.01 (extremely significant difference).

## 5. Conclusions

This study systematically identified TCP gene family members in three *Glycyrrhiza* species at the genome-wide level, clarified the positive regulatory role of the core salt-responsive gene *GuTCP16* in salt stress response, and provided candidate genes for salt-tolerant molecular breeding of licorice. The downstream target genes and specific molecular mechanism of *GuTCP16* still need further exploration via yeast one-hybrid and ChIP-seq assays. In particular, whether *GuTCP16* exerts its function through an ABA-dependent signaling pathway remains to be verified by quantifying endogenous ABA content and detecting the expression of ABA pathway marker genes. In addition, follow-up studies will systematically characterize the physiological and biochemical basis of *GuTCP16*-mediated salt tolerance: we will measure the activities of key antioxidant enzymes (SOD, POD, CAT), ROS accumulation levels, osmolyte contents (proline, soluble sugars) and ion homeostasis (Na^+^/K^+^ ratio) in plants with different genotypes under salt stress, to clarify the downstream physiological regulatory network of *GuTCP16*. On this basis, we will construct stably transformed *Glycyrrhiza* plants to verify its function under field conditions and explore its interactive regulatory network with other stress resistance genes.

## Figures and Tables

**Figure 1:**
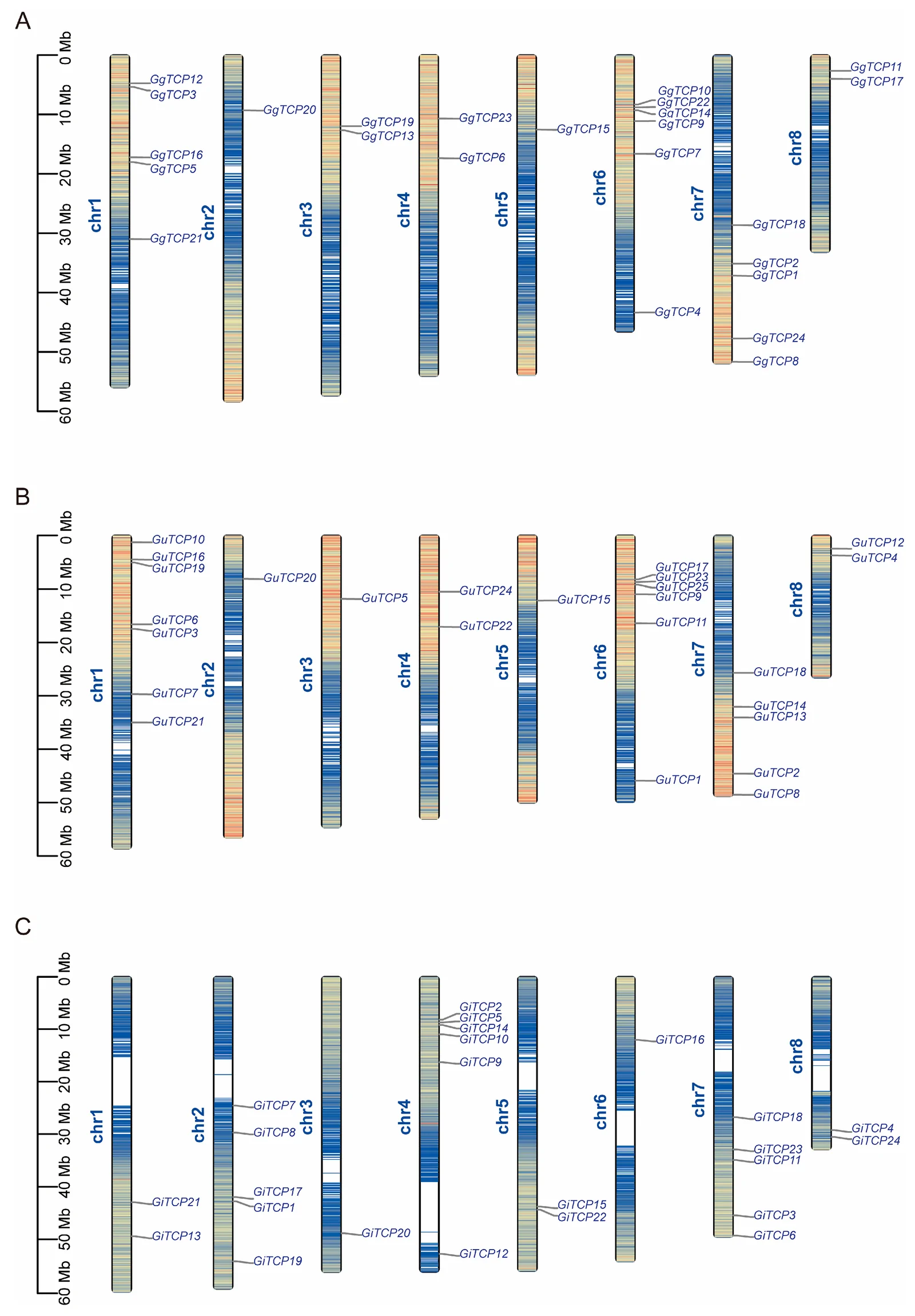
Chromosomal localization of TCP family genes in three *Glycyrrhiza* species. (**A**) Distribution of TCP genes across the chromosomes of *Glycyrrhiza* glabra (Gg); (**B**) Distribution of TCP genes across the chromosomes of *Glycyrrhiza* uralensis (Gu); (**C**) Distribution of TCP genes across the chromosomes of *Glycyrrhiza* inflata (Gi). The scale bar on the left indicates the chromosome length in megabases (Mb). Chromosome names are highlighted in blue, and individual TCP genes are labeled on the sides of each chromosome.The colors of the gene markers represent gene density: red indicates high gene density, and blue indicates low gene density.

**Figure 2:**
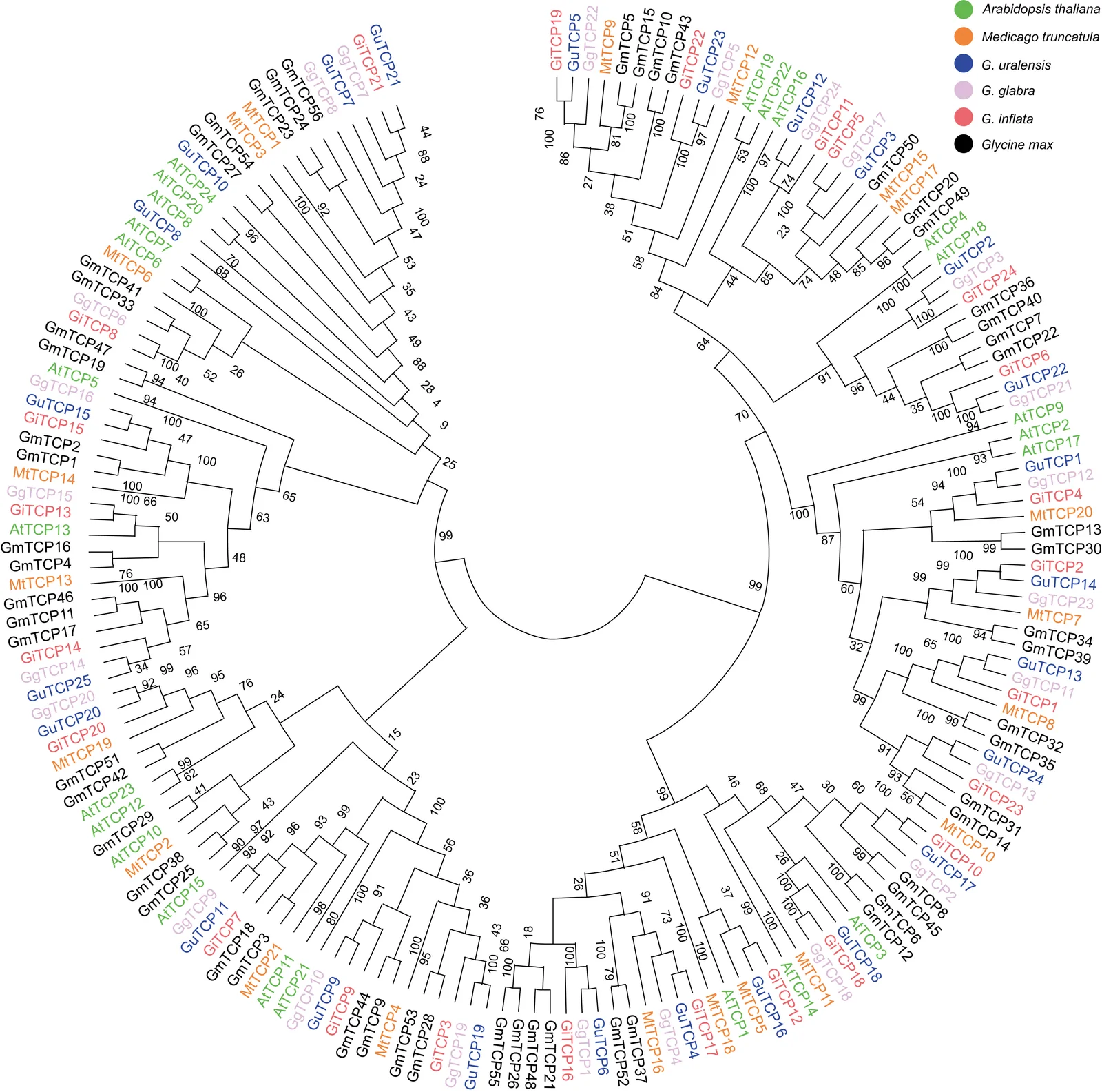
Phylogenetic analysis of TCP proteins from three *Glycyrrhiza* species and representative reference plants. The neighbor-joining (NJ) tree was constructed using full-length TCP protein sequences from *G. uralensis*, *G. glabra*, *G. inflata*, *Arabidopsis thaliana*, *Medicago truncatula* and *Glycine max*.

**Figure 3:**
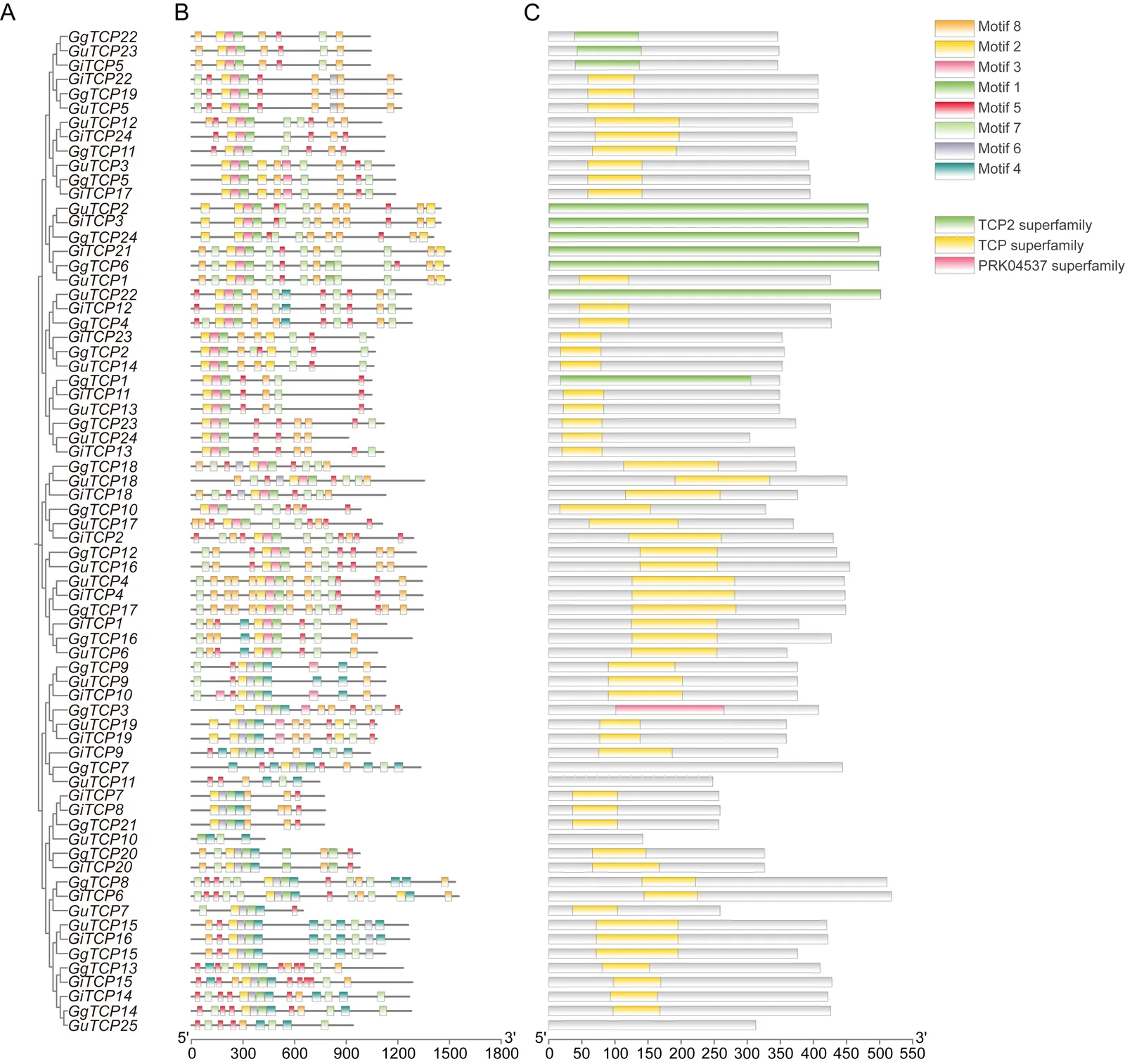
Sequence characteristics of TCP proteins in three *Glycyrrhiza* species. (**A**) Phylogenetic tree constructed with TCP proteins from the three *Glycyrrhiza* species; (**B**) Conserved motif analysis of TCP proteins; (**C**) Characterization of conserved functional domains in TCP proteins.

**Figure 4:**
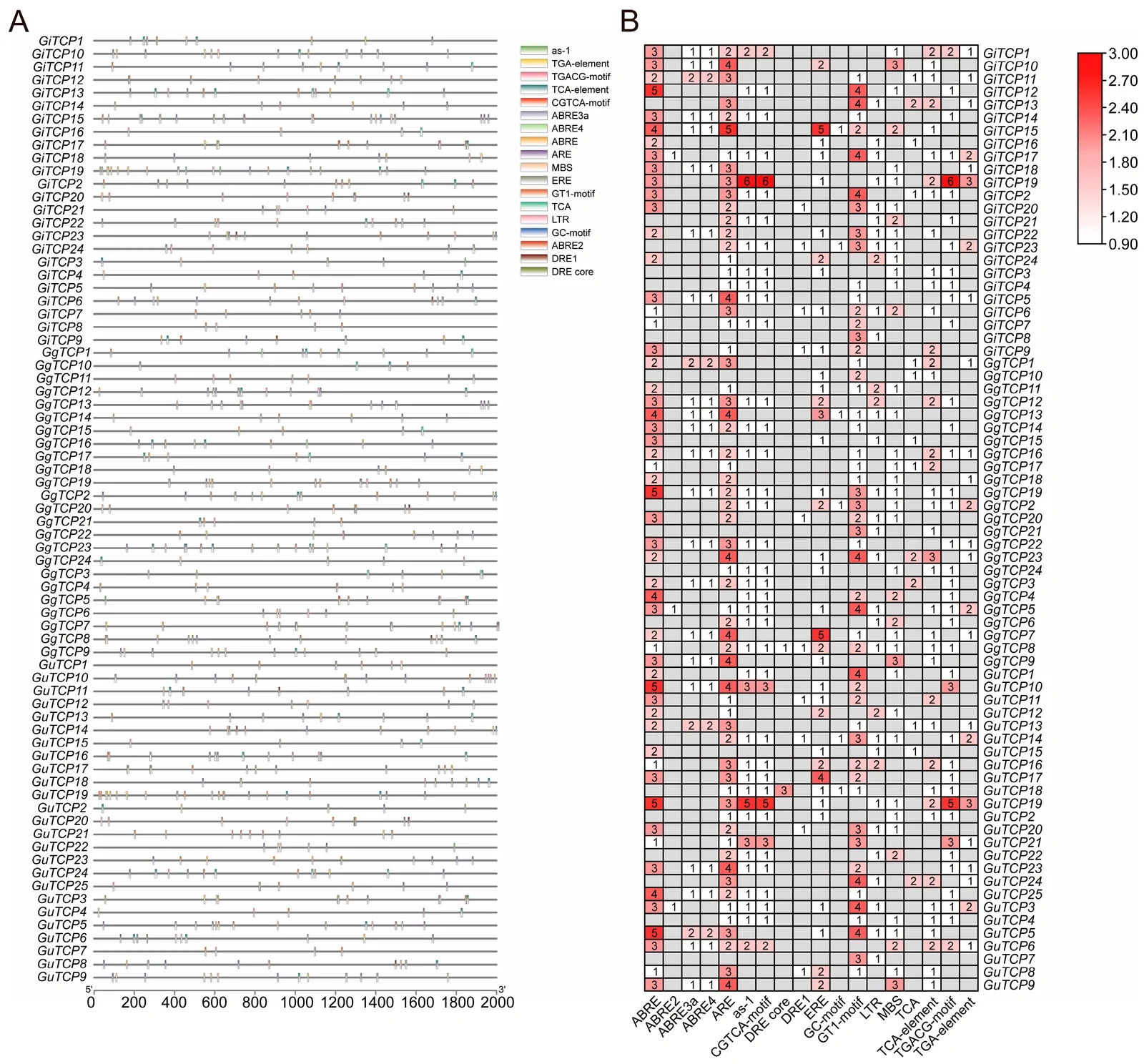
Analysis and prediction of cis-regulatory elements in the promoter regions (2000 bp upstream) of TCP genes in three *Glycyrrhiza* species. (**A**) Spatial organization of cis-regulatory elements within the promoter regions. Distinct functional motifs are annotated by color-coded boxes. (**B**) Heatmap profiling the abundance of cis-elements across TCP gene family members. The color gradient and numerical labels denote the specific counts of each element. These identified cis-regulatory elements are primarily associated with plant hormone responsiveness, abiotic stress responsiveness, and plant growth and development.

**Figure 5:**
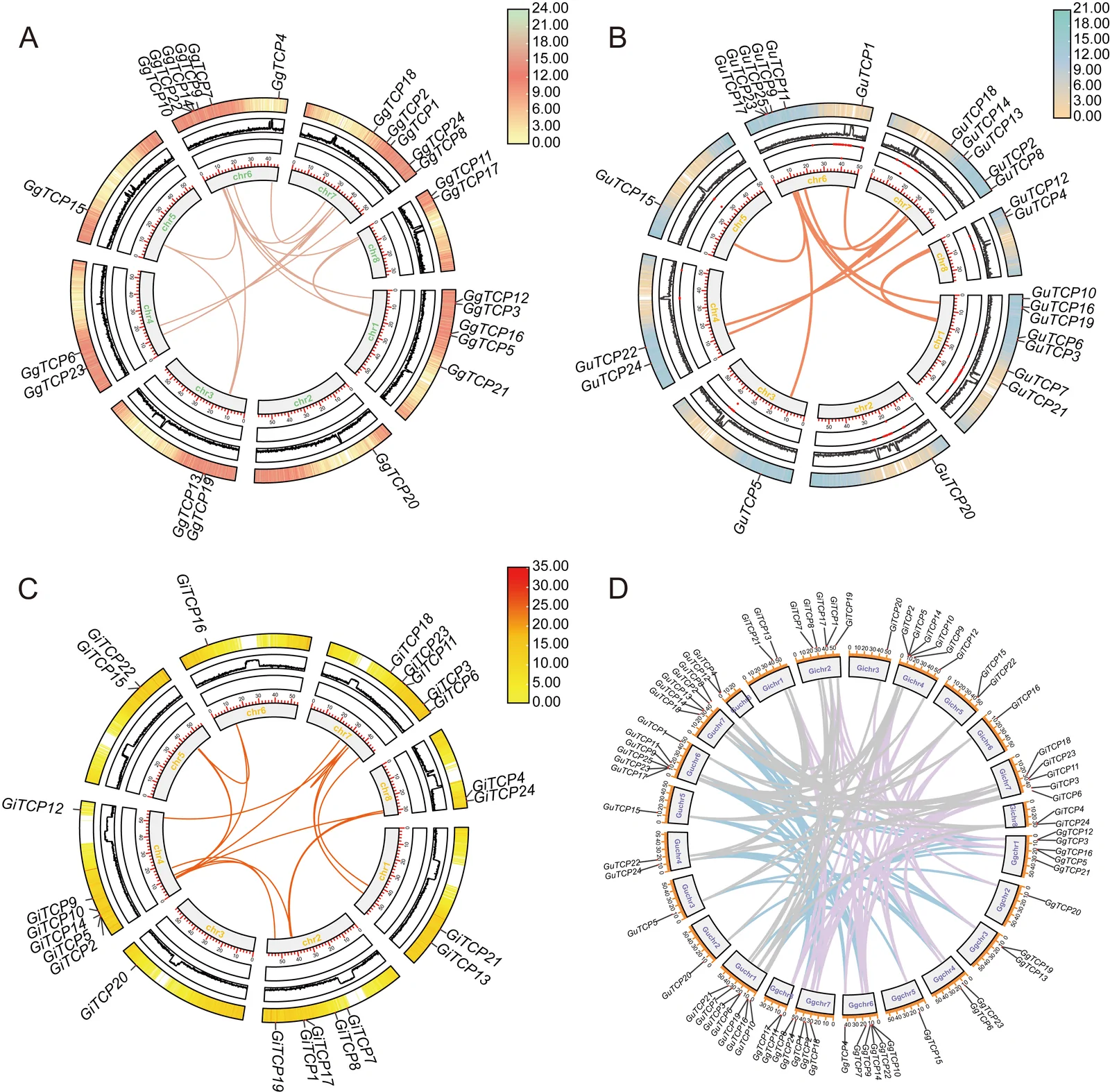
Synteny and segmental duplication analysis. (**A**–**C**) Intra-species collinearity analysis in *G. glabra*, *G. uralensis*, and *G. inflata*, showing segmental duplication events. The outermost circles represent gene density, while inner circles indicate CG content. Red curves represent collinear gene pairs. (**D**) Cross-species synteny analysis among the three *Glycyrrhiza* species, illustrating the orthologous relationships and evolutionary trajectories of the TCP gene family.

**Figure 6:**
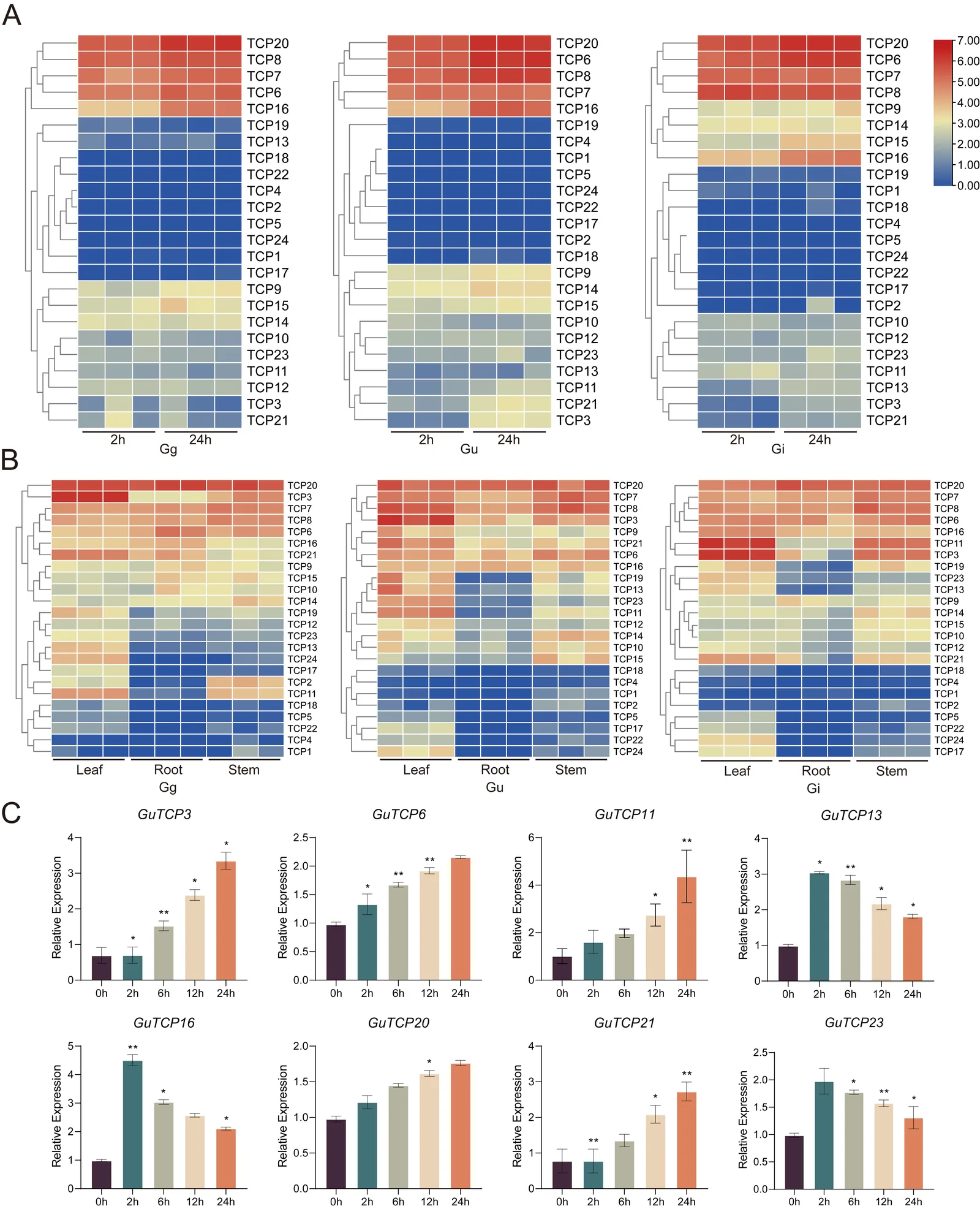
Expression specific to tissue analysis of the *Glycyrrhiza* TCP. (**A**) Transcriptomic analysis of TCP gene expression in three *Glycyrrhiza* species under salt stress (2 h and 24 h). (**B**) Tissue-specific expression patterns of TCP genes in roots, stems, and leaves. (**C**) qRT-PCR validation of representative TCP genes at various time points (0 h, 2 h, 6 h, 12 h, 24 h) under salt stress treatment (*n* = 3). The criteria for significant distinctions are as follows: *, *p* < 0.05 and **, *p* < 0.01.

**Figure 7:**
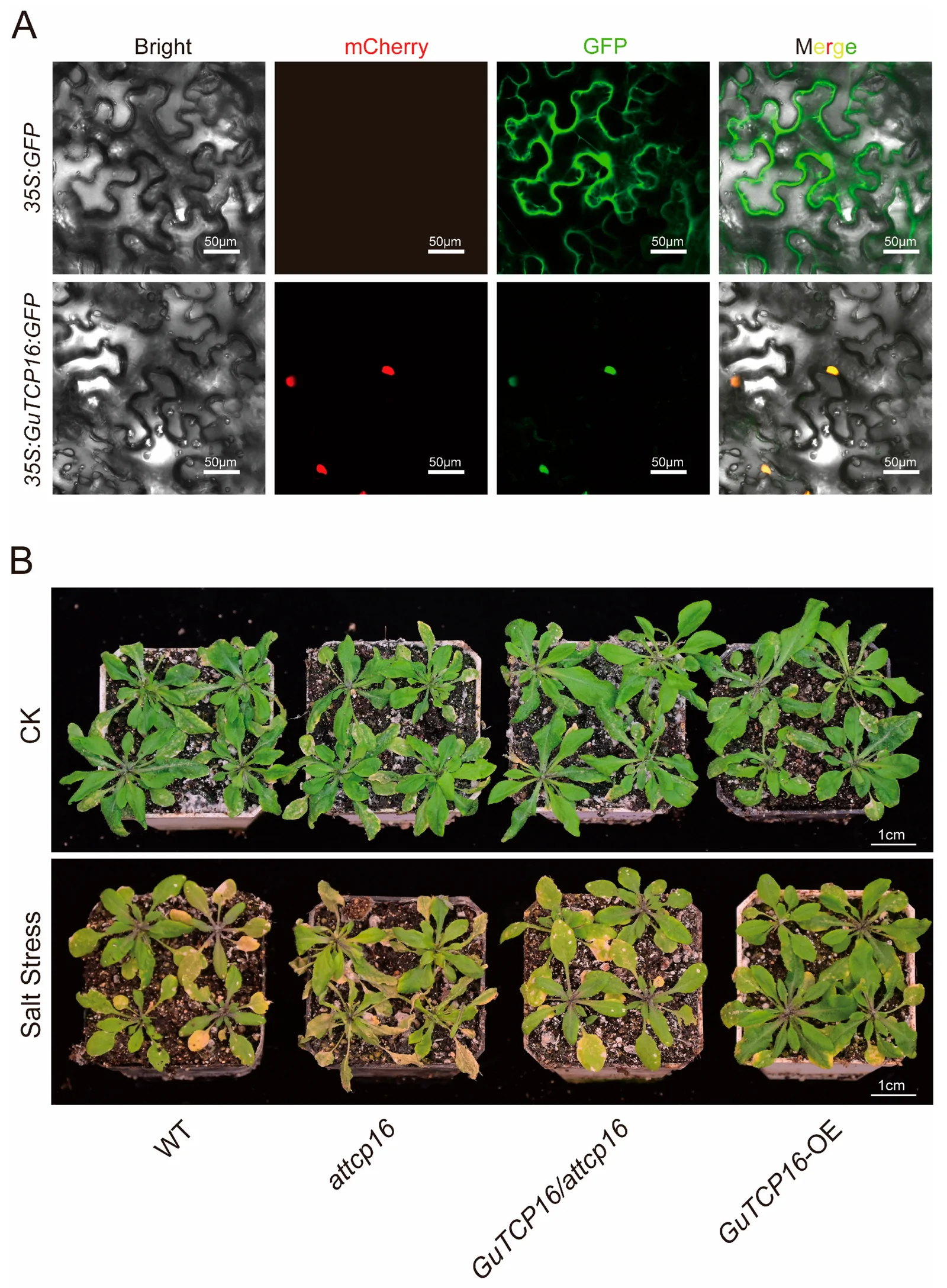
Subcellular localization and salt stress tolerance analysis of *GuTCP16*. (**A**) Subcellular localization of *GuTCP16*. The 35S: GFP construct served as a control, showing diffuse fluorescence throughout the cell. The *GuTCP16*-GFP fusion protein signals colocalize with the mCherry nuclear marker. Scale bar = 50 μm. (**B**) Phenotypic analysis of wild-type (WT), *attcp16* mutant, *GuTCP16*/*attcp16* complementation lines, and *GuTCP16*-OE (overexpression) lines under normal (CK) and salt stress conditions. Scale bar = 1 cm.

## Data Availability

Data are contained within the article and Supplementary Materials.

## Supplementary Materials

The following supporting information can be downloaded at: https://www.mdpi.com/article/10.3390/ijms27198446/s1.

## Abbreviations

The following abbreviations are used in this manuscript:

ABA	Abscisic Acid
ABRE	ABA-Responsive Element
ARE	Anaerobic Response Element
bHLH	basic Helix-Loop-Helix
GRAVY	Grand Average of Hydropathicity
LTR	Low-temperature Responsive Element
MBS	MYB Binding Site
MeJA	Methyl Jasmonate
SA	Salicylic Acid
TCP	TEOSINTE BRANCHED 1, CYCLOIDEA, PCF1/2
