# Supplementary material for: Genome-Wide Identification and Comparative Analysis of the TCP Transcription Factor Family in Three Glycyrrhiza Species Reveals GuTCP16 as a Positive Regulator of Salt Tolerance
Source: Int J Mol Sci. 2026 Sep 22;27(19):8446. doi: 10.3390/ijms27198446 (PMC13664917; doi:10.3390/ijms27198446)
Supplement: Supplementary file 1 [file ijms-27-08446-s001.zip › Supplementary Table S1.pdf]

Supplementary Table S1: Biochemical Characterization of TCP gene family members

Gene ID	Rename ID	Number of Amino Acid/aa	Molecular Weight / kDa	Theoretical pI	Instability Index	Aliphatic Index	Grand Average of Hydropathicity
GglaChr2G00031290	GgTCP20	325	34518.92	9.02	52	65.17	-0.78
GglaChr1G00087270	GgTCP21	256	27450.43	9.38	53.37	68.79	-0.614
GglaChr6G00256870	GgTCP14	425	46042.57	8	68.05	57.69	-0.792
GglaChr3G00053690	GgTCP13	409	44360.22	6.24	56.52	52.49	-0.863
GglaChr5G00174780	GgTCP15	375	39551.84	8.46	54.56	60.45	-0.531
GglaChr7G00229890	GgTCP8	510	54431.4	7.06	61.85	54.9	-0.773
GglaChr6G00254480	GgTCP9	375	39444.86	6.26	48.12	63.25	-0.45
GglaChr7G00203880	GgTCP18	373	41811.34	9.19	53.48	71.88	-0.621
GglaChr8G00283920	GgTCP17	448	50142.3	9.21	56.47	55.56	-0.951
GglaChr1G00098810	GgTCP16	426	48575.69	8.1	57.95	53.4	-1.018
GglaChr6G00258130	GgTCP10	327	37133.32	9.17	54.23	61.16	-0.981
GglaChr1G00113360	GgTCP12	434	48637.65	6.15	58.55	57.21	-0.735
GglaChr6G00231330	GgTCP4	426	44941.16	7.38	59.2	58.26	-0.634
GglaChr7G00208870	GgTCP2	355	39280.04	5.84	70.29	60.51	-0.79
GglaChr7G00210960	GgTCP1	348	37895.28	5.99	47	60.37	-0.747
GglaChr4G00132430	GgTCP23	372	41272.84	5.87	56.41	59.52	-0.844
GglaChr7G00224680	GgTCP24	468	51667.99	6.79	60.11	51.69	-0.997
GglaChr6G00257560	GgTCP22	345	38206.08	7.94	60.15	62.49	-0.739
GglaChr3G00052960	GgTCP19	406	44790.67	9.32	56.04	66.8	-0.634
GglaChr8G00285170	GgTCP11	372	41073.74	7.13	54.88	72.31	-0.574
GglaChr1G00097870	GgTCP5	394	43334.89	8.45	55.92	66.62	-0.766
GglaChr4G00140800	GgTCP6	498	54156.77	7.46	55.74	52.55	-0.876

GglaChr1G00112760	GgTCP3	407	43326.88	8.99	57.98	59.31	-0.637
GglaChr6G00248050	GgTCP7	443	46732.1	9.92	58.36	58.4	-0.584
Glycyrrhiza_uralensis_Fisch0048740.1	GuTCP20	324	34395.81	9.02	54.68	65.37	-0.77
Glycyrrhiza_uralensis_Fisch0033140.1	GuTCP21	256	27464.46	9.38	52.45	68.79	-0.614
Glycyrrhiza_uralensis_Fisch0031640.1	GuTCP7	258	27706.69	9.38	54.53	68.26	-0.636
Glycyrrhiza_uralensis_Fisch0267610.1	GuTCP8	215	22724.26	9.91	56.24	71.02	-0.46
Glycyrrhiza_uralensis_Fisch0006190.1	GuTCP19	358	38258.15	5.5	55.36	58.99	-0.621
Glycyrrhiza_uralensis_Fisch0174520.1	GuTCP15	419	43896.36	6.42	55.96	59.26	-0.579
Glycyrrhiza_uralensis_Fisch0207210.1	GuTCP9	375	39442.84	6.26	48.83	62.48	-0.465
Glycyrrhiza_uralensis_Fisch0241480.1	GuTCP18	450	50594.44	9.08	53.22	75.4	-0.562
Glycyrrhiza_uralensis_Fisch0272010.1	GuTCP4	446	49792.96	9.14	56	57.13	-0.926
Glycyrrhiza_uralensis_Fisch0020000.1	GuTCP6	359	40557.22	6.73	54.7	45.46	-1.136
Glycyrrhiza_uralensis_Fisch0203500.1	GuTCP17	369	41893.45	8.27	52.28	60.57	-0.985
Glycyrrhiza_uralensis_Fisch0005610.1	GuTCP16	454	51009.27	6.38	58.55	55.77	-0.748
Glycyrrhiza_uralensis_Fisch0231350.1	GuTCP1	425	44937.24	7.38	59.27	60	-0.616
Glycyrrhiza_uralensis_Fisch0246540.1	GuTCP14	352	38877.65	5.92	66.95	59.91	-0.781
Glycyrrhiza_uralensis_Fisch0248710.1	GuTCP13	348	37864.31	5.99	47.77	61.78	-0.72
Glycyrrhiza_uralensis_Fisch0132630.1	GuTCP24	303	33971.44	5.99	49.77	65.31	-0.76
Glycyrrhiza_uralensis_Fisch0262340.1	GuTCP2	482	53337.83	6.9	62.25	51.8	-1.023
Glycyrrhiza_uralensis_Fisch0204110.1	GuTCP23	347	38304.19	7.95	58.96	62.13	-0.751
Glycyrrhiza_uralensis_Fisch0270930.1	GuTCP12	367	40592.24	7.44	51.41	70.11	-0.615
Glycyrrhiza_uralensis_Fisch0095640.1	GuTCP5	406	44762.65	9.3	55.31	66.8	-0.632
Glycyrrhiza_uralensis_Fisch0021020.1	GuTCP3	392	43094.63	7.91	54.15	66.71	-0.756
Glycyrrhiza_uralensis_Fisch0140980.1	GuTCP22	501	54478.19	7.46	56.56	53.21	-0.867
Glycyrrhiza_uralensis_Fisch0001530.1	GuTCP10	141	15120.03	11.03	73.52	70.14	-0.419
Glycyrrhiza_uralensis_Fisch0213770.1	GuTCP11	247	25788.09	7.86	67.29	71.17	-0.26

Glycyrrhiza_uralensis_Fisch0204820.1	GuTCP25	312	33682.48	6.26	69.92	52.31	-0.87
GuraChr3G111760	GiTCP20	325	34488.9	9.02	51.15	65.17	-0.779
GuraChr2G074380	GiTCP19	358	38272.18	5.5	56.14	59.27	-0.615
GuraChr2G047800	GiTCP7	256	27450.43	9.38	53.37	68.79	-0.614
GuraChr2G049070	GiTCP8	258	27736.72	9.38	54.53	67.87	-0.646
GuraChr4G138160	GiTCP9	345	36369.19	9.72	62.12	72.52	-0.338
GuraChr4G129190	GiTCP14	421	45361.8	7.41	70.07	57.55	-0.77
GuraChr5G178940	GiTCP15	427	46434.46	6.39	57.14	52.55	-0.884
GuraChr7G261030	GiTCP6	517	55104.18	7.06	61.8	54.55	-0.776
GuraChr6G208380	GiTCP16	421	44053.49	6.42	55.38	58.74	-0.586
GuraChr4G131530	GiTCP10	375	39416.8	6.26	48.32	62.75	-0.456
GuraChr7G234850	GiTCP18	375	41981.46	9.11	56.22	71.49	-0.623
GuraChr8G275950	GiTCP4	447	49930.1	9.14	55.37	57	-0.931
GuraChr2G060570	GiTCP1	377	42592.48	6.46	55.95	46.39	-1.098
GuraChr4G127810	GiTCP2	429	48588.68	7.31	52.91	57.55	-0.94
GuraChr4G155230	GiTCP12	425	44961.22	7.39	58.82	59.08	-0.633
GuraChr7G239740	GiTCP23	352	38876.67	6	66.4	59.91	-0.781
GuraChr7G241940	GiTCP11	348	37864.31	5.99	47.22	61.78	-0.718
GuraChr1G027040	GiTCP13	371	41193.78	5.87	55.9	59.68	-0.831
GuraChr7G255680	GiTCP3	482	53337.83	6.9	62.25	51.8	-1.023
GuraChr4G128450	GiTCP5	345	38148.08	8.51	59.33	62.49	-0.735
GuraChr8G277050	GiTCP24	374	41219.76	7.13	54.64	68.8	-0.611
GuraChr2G059640	GiTCP17	394	43324.86	8.45	56.05	66.62	-0.764
GuraChr5G179700	GiTCP22	406	44763.64	9.32	56.04	66.8	-0.627
GuraChr1G018710	GiTCP21	501	54476.26	7.83	56.25	53.99	-0.851
