# Supplementary material for: Genome-Wide Identification and Comparative Analysis of the TCP Transcription Factor Family in Three Glycyrrhiza Species Reveals GuTCP16 as a Positive Regulator of Salt Tolerance
Source: Int J Mol Sci. 2026 Sep 22;27(19):8446. doi: 10.3390/ijms27198446 (PMC13664917; doi:10.3390/ijms27198446)
Supplement: Supplementary file 1 [file ijms-27-08446-s001.zip › Supplementary Table S2.pdf]

Supplementary Table S2: Primers used in this experiment

Primer name	Sequence (from 5′ to 3′)
GuTCP20-F-QPCR	TCATGTGGCCACTTCTGGTC
GuTCP20-R-QPCR	AACCCCAGTTCCAAACCAGG
GuTCP21-F-QPCR	TAACGACCAAGACGACACCG
GuTCP21-R-QPCR	AAGCTTCTTGTTGCTGCTGC
GuTCP6-F-QPCR	CAACACCCTTGAGTGGCTCT
GuTCP6-R-QPCR	CCTTTCCCTTGCTCTAGCCC
GuTCP16-F-QPCR	AGGCTTTCGAGCGAGATAGC
GuTCP16-R-QPCR	ACCAGAAGCTGCTGCATCTT
GuTCP13-F-QPCR	CAGCAAAGGGTCCTCGTGAT
GuTCP13-R-QPCR	TTTGAGTTGGGTGCCATGGT
GuTCP23-F-QPCR	AATTCGAGGCAAGAGGGTGG
GuTCP23-R-QPCR	AACCTTGCTGGGTTGGTTGA
GuTCP3-F-QPCR	GCATCATGTCCTCCTCCTGG
GuTCP3-R-QPCR	GGCAGGTGAATGGAGAAGCT
GuTCP11-F-QPCR	AAGAAGAAACGCAAACGGCC
GuTCP11-R-QPCR	GCTGTGTTAGGGACCACGAA
GuTCP16-F	ATGCAGTTCTCTTCCACATATCATGAC
GuTCP16-R	GTTGTGTCGACTCTGATTGTTTCGC
GuTCP16-F-1300	atttggagaggacagggtaccATGCAGTTCTCTTCCACATATCATGAC
GuTCP16-R-1300	caccatggtactagtgtcgacGTTGTGTCGACTCTGATTGTTTCGC
