# Supplementary material for: Genome-Wide Identification and Comparative Analysis of the TCP Transcription Factor Family in Three Glycyrrhiza Species Reveals GuTCP16 as a Positive Regulator of Salt Tolerance
Source: Int J Mol Sci. 2026 Sep 22;27(19):8446. doi: 10.3390/ijms27198446 (PMC13664917; doi:10.3390/ijms27198446)
Supplement: Supplementary file 1 [file ijms-27-08446-s001.zip › Supplementary Table S3.pdf]

Supplementary Table S3:The Connection between the Database and the Online Website in This Study

TAIR	https://www.arabidopsis.org, accessed on 10 Apr 2026
InterProScan	https://www.ebi.ac.uk/interpro/about/interproscan/, accessed on 12 Apr, 2026
SMART	https://smart.embl.de/, accessed on 12 Apr, 2026
ExPASY	https://web.expasy.org/compute_pi/, accessed on 13 Apr 2026
iTOL	https://itol.embl.de/, accessed on 13 Apr 2026
MG2C_v2.1	http://mg2c.iask.in/mg2c_v2.1/, accessed on 12 Apr 2026
MEME	https://meme-suite.org/meme/tools/meme, accessed on 13 Apr 2026
CD-Search	https://www.ncbi.nlm.nih.gov/Structure/cdd/wrpsb.cgi, accessed on 14 Apr 2026
PlantCARE	https://bioinformatics.psb.ugent.be/webtools/plantcare/html/, accessed on 15 Apr 2026
