# Supplementary material for: Genome-Wide Identification and Comparative Analysis of the TCP Transcription Factor Family in Three Glycyrrhiza Species Reveals GuTCP16 as a Positive Regulator of Salt Tolerance
Source: Int J Mol Sci. 2026 Sep 22;27(19):8446. doi: 10.3390/ijms27198446 (PMC13664917; doi:10.3390/ijms27198446)
Supplement: Supplementary file 1 [file ijms-27-08446-s001.zip › Supplementary Table S4.pdf]

TableS4: The sequence information for the Arabidopsis TCP proteins

>AT5G41030.1
MVMEPKKNQNLPSFLNPSRQNQDNDKKRKQTEVKGFDIVVGEKRKKKENEEE
DQEIQILYEKEKKKPNKDRHLKVEGRGRRVRLPPLCAARIYQLTKELGHKSDGE
TLEWLLQHAEPSILSATVNGIKPTESVVSQPPLTADLMICHSVEEASRTQMEANG
LWRNETGQTIGGFDLNYGIGFDFNGVPEIGFGDNQTPGLELRLSQVGVLNPQVF
QQMGKEQFRVLHHHSHEDQQQSAEENGS*

>AT1G68800.2
MFPSLDTNGYDLFDPFIPHQTTMFPSFITHIQSPNSHHHYSSPSFPFSSDFLESFDE
SFLINQFLLQQQDVAANVVESPWKFCKKLELKKKNEKCVDGSTSQEVQWRRTV
KKRDRHSKICTAQGPRDRRMRLSLQIARKFFDLQDMLGFDKASKTIEWLFSKSK
TSIKQLKERVAASEGGGKDEHLQVDEKEKDETLKLRVSKRRTKTMESSFKTKESR
ERARKRARERTMAKMKMRLFETSETISDPHQETREIKITNGVQLLEKENKEQEW
SNTNDVHMVEYQMDSVSIIEKFLGLTSDSSSSSIFGDSEECYTSLSSVRGTISAAGM
STPREHNTTSIATVDEEKSPISSFSLYDYLCY*
>AT1G72010.1
MNQNSSVAEATLQLNSGEKPSPGSIPFISSGQHGNISTSATSSTSTSSGSALAVVKS
AVKKPTKDRHTKVDGRGRRIRMPAMCAARVFQLTRELGHKSDGETIEWLLQQ
AEPAIIASTGTGTIPANFSTLNASLRSGGGSTLFSQASKSSSSPLSFHSTGMSLYED
NNGTNGSSVDPSRKLLNSAANAAVFGFHHQMYPPIMSTERNPNTLVKPYREDY
FKEPSSAAEPSESSQKASQFQEQELAQGRGTANVVPQPMWAVAPGTTNGGSAF
WMLPMSGSGGREQMQQQPGHQMWAFNPGNYPVGTGRVVTAPMGSMMLGG
QQLGLGVAEGNMAAAMRGSRGDGLAMTLDQHQHQLQHQEPNQSQASENG
GDDKK*
>AT5G60970.1
MRSGECDEEEIQAKQERDQNQNHQVNLNHMLQQQQPSSVSSSRQWTSAFRNP
RIVRVSRTFGGKDRHSKVCTVRGLRDRRIRLSVPTAIQLYDLQDRLGLSQPSKVID
WLLEAAKDDVDKLPPLQFPHGFNQMYPNLIFGNSGFGESPSSTTSTTFPGTNLG
FLENWDLGGSSRTRARLTDTTTTQRESFDLDKGKWIKNDENSNQDHQGFNTN
HQQQFPLTNPYNNTSAYYNLGHLQQSLDQSGNNVTVAISNVAANNNNNLNL
HPPSSSAGDGSQLFFGPTPPAMSSLFPTYPSFLGASHHHHVVDGAGHLQLFSSNS
NTASQQHMMPGNTSLIRPFHHLMSSNHDTDHHSSDNESDS*
>AT1G69690.1
MDPDPDHNHRPNFPLQLLDSSTSSSSTSLAIISTTSEPNSEPKKPPPKRTSTKDRHT
KVEGRGRRIRMPAMCAARVFQLTRELGHKSDGETIEWLLQQAEPAVIAATGTG
TIPANFTSLNISLRSSRSSLSAAHLRTTPSSYYFHSPHQSMTHHLQHQHQVRPKN
ESHSSSSSSSQLLDHNQMGNYLVQSTAGSLPTSQSPATAPFWSSGDNTQNLWAF
NINPHHSGVVAGDVYNPNSGGSGGGSGVHLMNFAAPIALFSGQPLASGYGGG
GGGGGEHSHYGVLAALNAAYRPVAETGNHNNNQQNRDGDHHHNHQEDGS
TSHHS*
>AT1G67260.1
MSSSTNDYNDGNNNGVYPLSLYLSSLSGHQDIIHNPYNHQLKASPGHMVSAVP
ESLIDYMAFKSNNVVNQQGFEFPEVSKEIKKVVKKDRHSKIQTAQGIRDRRVRLS
IGIARQFFDLQDMLGFDKASKTLDWLLKKSRKAIKEVVQAKNLNNDDEDFGNI

GGDVEQEEEKEEDDNGDKSFVYGLSPGYGEEEVVCEATKAGIRKKKSELRNISS
KGLGAKARGKAKERTKEMMAYDNPETASDITQSEIMDPFKRSIVFNEGEDMTH
LFYKEPIEEFDNQESILTNMTLPTKMGQSYNQNNGILMLVDQSSSSNYNTFLPQ
NLDYSYDQNPFHDQTLYVVTDKNFPKGKVWIQDSFVN*
>AT5G08330.1
MADNDGAVSNGIIVEQTSNKGPLNAVKKPPSKDRHSKVDGRGRRIRMPIICAA
RVFQLTRELGHKSDGQTIEWLLRQAEPSIIAATGTGTTPASFSTASLSTSSPFTLGK
RVVRAEEGESGGGGGGGLTVGHTMGTSLMGGGGSGGFWAVPARPDFGQVWS
FATGAPPEMVFAQQQQPATLFVRHQQQQQASAAAAAAMGEASAARVGNYL
PGHHLNLLASLSGGANGSGRREDDHEPR*
>AT5G51910.1
MESNHEGNAIQVIDQVTTMTHLSDPNPKTKPGMMLMKQEDGYLQPVKTKPAP
KRPTSKDRHTKVEGRGRRIRMPAGCAARVFQLTRELGHKSDGETIRWLLERAEP
AIIEATGTGTVPAIAVSVNGTLKIPTSSPVLNDGGRDGDGDLIKKRRKRNCTSDF
VDVNDSCHSSVTSGLAPITASNYGVNILNVNTQGFVPFWPMGMGTAFVTGGPD
QMGQMWAIPTVATAPFLNVGARPVSSYVSNASDAEAEMETSGGGTTQPLRDFS
LEIYDKRELQFLGGSGNSSPSSCHET*
>AT3G47620.1
MQKPTSSILNVIMDGGDSVGGGGGDDHHRHLHHHHRPTFPFQLLGKHDPDD
NHQQQPSPSSSSSLFSLHQHQQLSQSQPQSQSQKSQPQTTQKELLQTQEESAVV
AAKKPPLKRASTKDRHTKVDGRGRRIRMPALCAARVFQLTRELGHKSDGETIE
WLLQQAEPSVIAATGTGTIPANFTSLNISLRSSGSSMSLPSHFRSAASTFSPNNIFS
PAMLQQQQQQQRGGGVGFHHPHLQGRAPTSSLFPGIDNFTPTTSFLNFHNPTK
QEGDQDSEELNSEKKRRIQTTSDLHQQQQQHQHDQIGGYTLQSSNSGSTATAA
AAQQIPGNFWMVAAAAAAGGGGGNNNQTGGLMTASIGTGGGGGEPVWTFP
SINTAAAALYRSGVSGVPSGAVSSGLHFMNFAAPMAFLTGQQQLATTSNHEIN
EDSNNNEGGRSDGGGDHHNTQRHHHHQQHHHNILSGLNQYGRQVSGDSQ
ASGSLGGGDEEDQQD*
>AT3G15030.1
MSDDQFHHPPPPSSMRHRSTSDAADGGCGEIVEVQGGHIVRSTGRKDRHSKVC
TAKGPRDRRVRLSAHTAIQFYDVQDRLGFDRPSKAVDWLIKKAKTSIDELAELP
PWNPADAIRLAAANAKPRRTTAKTQISPSPPPPQQQQQQQQLQFGVGFNGGG
AEHPSNNESSFLPPSMDSDSIADTIKSFFPVIGSSTEAPSNHNLMHNYHHQHPPD
LLSRTNSQNQDLRLSLQSFPDGPPSLLHHQHHHHTSASASEPTLFYGQSNPLGF
DTSSWEQQSSEFGRIQRLVAWNSGGGGGATDTGNGGGFLFAPPTPSTTSFQPVL
GQSQQLYSQRGPLQSSYSPMIRAWFDPHHHHQSISTDDLNHHHHHLPPPVHQSA
IPGIGFASGEFSSGFRIPARFQGQEEEQHDGLTHKPSSASSISRH*
>AT3G27010.1
MDPKNLNRHQVPNFLNPPPPPRNQGLVDDDAASAVVSDENRKPTTEIKDFQIV
VSASDKEPNKKSQNQNQLGPKRSSNKDRHTKVEGRGRRIRMPALCAARIFQLT
RELGHKSDGETIQWLLQQAEPSIIAATGSGTIPASALASSAATSNHHQGGSLTAG
LMISHDLDGGSSSSGRPLNWGIGGGEGVSRSSLPTGLWPNVAGFGSGVPTTGLM
SEGAGYRIGFPGFDFPGVGHMSFASILGGNHNQMPGLELGLSQEGNVGVLNPQ
SFTQIYQQMGQAQAQAQGRVLHHMHHNHEEHQQESGEKDDSQGSGR*

>AT4G18390.1
MIGDLMKNNNNGDVVDNEVNNRLSRWHHNSSRIIRVSRASGGKDRHSKVLTS
KGPRDRRVRLSVSTALQFYDLQDRLGYDQPSKAVEWLIKAAEDSISELPSLNNT
HFPTDDENHQNQTLTTVAANSLSKSACSSNSDTSKNSSGLSLSRSELRDKARER
ARERTAKETKERDHNHTSFTDLLNSGSDPVNSNRQWMASAPSSSPMEYFSSGLI
LGSGQQTHFPISTNSHPFSSISDHHHHHPHHQHQEFSFVPDHLISPAESNGGAF
NLDFNMSTPSGAGAAVSAASGGGFSGFNRGTLQSNSTNQHQSFLANLQRFPTS
ESGGGPQFLFGALPAENHHHNHQFQLYYENGCRNSSEHKGKGKN*
>AT5G23280.1
MSINNNNNNNNNNNNDGLMISSNGALIEQQPSVVVKKPPAKDRHSKVDGRGR
RIRMPIICAARVFQLTRELGHKSDGQTIEWLLRQAEPSIIAATGTGTTPASFSTASV
SIRGATNSTSLDHKPTSLLGGTSPFILGKRVRADEDSNNSHNHSSVGKDETFTTT
PAGFWAVPARPDFGQVWSFAGAPQEMFLQQQHHHQQPLFVHQQQQQQAA
MGEASAARVGNYLPGHLNLLASLSGGSPGSDRREEDPR*
>AT5G08070.1
MGIKKEDQKSSLSLLTQRWNNPRIVRVSRAFGGKDRHSKVCTVRGLRDRRIRLS
VMTAIQVYDLQERLGLSQPSKVIDWLLEVAKNDVDLLPPLQFPPGFHQLNPNL
TGLGESFPGVFDLGRTQREALDLEKRKWVNLDHVFDHIDHHNHFSNSIQSNKL
YFPTITSSSSSYHYNLGHLQQSLLDQSGNVTVAFSNNYNNNNLNPPAAETMSSL
FPTRYPSFLGGGQLQLFSSTSSQPDHIE*
>AT2G45680.1
MATIQKLEEVAGKDQTLRAVDLTIINGVRNVETSRPFQVNPTVSLEPKAEPVMP
SFSMSLAPPSSTGPPLKRASTKDRHTKVEGRGRRIRMPATCAARIFQLTRELGHK
SDGETIRWLLENAEPAIIAATGTGTVPAIAMSVNGTLKIPTTTNADSDMGENLM
KKKRKRPSNSEYIDISDAVSASSGLAPIATTTTIQPPQALASSTVAQQLLPQGMYP
MWAIPSNAMIPTVGAFFLIPQIAGPSNQPQLLAFPAAAASPSSYVAAVQQASTM
ARPPPLQVVPSSGFVSVSDVSGSNLSRATSVMAPSSSSGVTTGSSSSIATTTTHTLR
DFSLEIYEKQELHQFMSTTTARSSNH*
>AT2G31070.1
MGLKGYSVGEGGGEIVEVQGGHIIRATGRKDRHSKVFTSKGPRDRRVRLSAHTA
IQFYDVQDRLGYDRPSKAVDWLIKKAKTAIDKLELGETTTTTTRQEPVNTKPESP
TLVFQRENNDQTQFVAANLDPEDAMKTFFPATTTTNGGGGTNINFQNYPHQD
DNNMVSRTTTPPPNLSQDLGLSLHPFQGNNNTVVVPETNNFTTTHFDTFGRISG
WNHHDLTMTSSSSSEHQQQEQEERSNGGFMVNHHPHHHHHQPSMMTLLNS
QQQQVFLGGQQQQQQRGTLQSSLFPHSFRSWDHHQTTSDHHHHQNQASSMF
ASSSQYGSHGMMMMQGLSFPNTTRLLHGEEATQPNSSSSPPNSHL*
>AT3G18550.3
MNNNIFSTTTTINDDYMLFPYNDHYSSQPLLPFSPSSSINDILIHSTSNTSNNHLD
HHHQFQQPSPFSHFEFAPDCALLTSFHPENNGHDDNQTIPNDNHHPSLHFPLN
NTIVEQPTEPSETINLIEDSQRISTSQDPKMKKAKKPSRTDRHSKIKTAKGTRDRR
MRLSLDVAKELFGLQDMLGFDKASKTVEWLLTQAKPEIIKIATTLSHHGCFSSG
DESHIRPVLGSMDTSSDLCELASMWTVDDRGSNTNTTETRGNKVDGRSMRGKR
KRPEPRTPILKKLSKEERAKARERAKGRTMEKMMMKMKGRSQLVKVVEEDAH

DHGEIIKNNNRSQVNRSSFEMTHCEDKIEELCKNDRFAVCNEFIMNKKDHISNE
SYDLVNYKPNSSFPVINHHRSQGAANSIEVPYLYLRVTFIIFMSFFPMVLSKAIHS*
>AT3G45150.1
MDSKNGINNSQKARRTPKDRHLKIGGRDRRIRIPPSVAPQLFRLTKELGFKTDGE
TVSWLLQNAEPAIFAATGHGVTTTSNEDIQPNRNFPSYTFNGDNISNNVFPCTV
VNTGHRQMVFPVSTMTDHAPSTNYSTISDNYNSTFNGNATASDTTSAATTTAT
TTV*
>AT3G02150.2
MNIVSWKDANDEVAGGATTRREREVKEDQEETEVRATSGKTVIKKQPTSISSSS
SWMKSKDPRIVRVSRAFGGKDRHSKVCTLRGLRDRRVRLSVPTAIQLYDLQERL
GVDQPSKAVDWLLDAAKEEIDELPPLPISPENFSIFNHHQSFLNLGQRPGQDPT
QLGFKINGCVQKSTTTSREENDREKGENDVVYTNNHHVGSYGTYHNLEHHHH
HHQHLSLQADYHSHQLHSLVPFPSQILVCPMTTSPTTTTIQSLFPSSSSAGSGTME
TLDPRQMVSHFQMPLMGNSSSSSSQNISTLYSLLHGSSSNNGGRDIDNRMSSVQ
FNRTNSTTTANMSRHLGSERCTSRGSDHHM*
>AT1G58100.1
MDLSDIRNNNNDTAAVATGGGARQLVDASLSIVPRSTPPEDSTLATTSSTATAT
TTKRSTKDRHTKVDGRGRRIRMPALCAARVFQLTRELGHKSDGETIEWLLQQA
EPAIVAATGTGTIPANFSTLSVSLRSSGSTLSAPPSKSVPLYGALGLTHHQYDEQG
GGGVFAAHTSPLLGFHHQLQHHQNQNQNQDPVETIPEGENFSRKRYRSVDLS
KENDDRKQNENKSLKESETSGPTAAPMWAVAPPSRSGAGNTFWMLPVPTTAG
NQMESSSNNNTAAGHRAPPMWPFVNSAGGGAGGGGGAATHFMAGTGFSFP
MDQYRGSPLQLGSFLAQPQPTQNLGLSMPDSNLGMLAALNSAYSRGGNANA
NAEQANNAVEHQEKQQQSDHDDDSREENSNSSE*
>AT2G37000.1
MIFQNVCRNESNFNAIASESRSQTQFGVSKSSSSGGGCISARTKDRHTKVNGRSR
RVTMPALAAARIFQLTRELGHKTEGETIEWLLSQAEPSIIAATGYGTKLISNWVD
VAADDSSSSSSMTSPQTQTQTPQSPSCRLDLCQPIGIQYPVNGYSHMPFTAMLLE
PMTTTAESEVEIAEEEERRRRHH*
>AT1G53230.1
MAPDNDHFLDSPSPPLLEMRHHQSATENGGGCGEIVEVQGGHIVRSTGRKDR
HSKVCTAKGPRDRRVRLSAPTAIQFYDVQDRLGFDRPSKAVDWLITKAKSAIDD
LAQLPPWNPADTLRQHAAAAANAKPRKTKTLISPPPPQPEETEHHRIGEEEDN
ESSFLPASMDSDSIADTIKSFFPVASTQQSYHHQPPSRGNTQNQDLLRLSLQSFQ
NGPPFPNQTEPALFSGQSNNQLAFDSSTASWEQSHQSPEFGKIQRLVSWNNVG
AAESAGSTGGFVFASPSSLHPVYSQSQLLSQRGPLQSINTPMIRAWFDPHHHHH
HHQQSMTTDDLHHHHPYHIPPGIHQSAIPGIAFASSGEFSGFRIPARFQGEQEEH
GGDNKPSSASSDSRH*
>AT1G35560.1
MESHNNNQSNNNTTGSAHLVPSMGPISGSVSLTTTAPNSTTTTVTAAKTPAKRP
SKDRHIKVDGRGRRIRMPAICAARVFQLTRELQHKSDGETIEWLLQQAEPAIIAA
TGTGTIPANISTLNISLRSSGSTLSAPLSKSFHMGRAAQNAAVFGFQQQLYHPHH
ITTDSSSSSLPKTFREEDLFKDPNFLDQEPGSRSPKPGSEAPDQDPGSTRSRTQNMI
PPMWALAPTPASTNGGSAFWMLPVGGGGGPANVQDPSQHMWAFNPGHYPG

RIGSVQLGSMLVGGQQLGLGVAENNNLGLFSGGGGDGGRVGLGMSLEQKPQ
HQVSDHATRDQNPTIDGSP*
>AT1G30210.1
MEVDEDIELQKHQEQQSRKLQRFSEDNTGLMRNWNNPSSRIIRVSRASGGKDR
HSKVLTSKGLRDRRIRLSVATAIQFYDLQDRLGFDQPSKAVEWLINAASDSITDL
PLLNTNFDHLDQNQNQTKSACSSGTSESSLLSLSRTEIRGKARERARERTAKDRD
KDLQNAHSSFTQLLTGGFDQQPSNRNWTGGSDCFNPVQLQIPNSSSQEPMNHP
FSFVPDYNFGISSSSSAINGGYSSRGTLQSNSQSLFLNNNNNITQRSSISSSSSSSP
MDSQSISFFMATPPPLDHHNHQLPETFDGRLYLYYGEGNRSSDDKAKERR*
