# Supplementary material for: Genome-Wide Identification and Comparative Analysis of the TCP Transcription Factor Family in Three Glycyrrhiza Species Reveals GuTCP16 as a Positive Regulator of Salt Tolerance
Source: Int J Mol Sci. 2026 Sep 22;27(19):8446. doi: 10.3390/ijms27198446 (PMC13664917; doi:10.3390/ijms27198446)
Supplement: Supplementary file 1 [file ijms-27-08446-s001.zip › Supplementary Table S5.pdf]

Table S5 Ka/Ks analysis of segmental duplicated TCP gene pairs in three *Glycyrrhiza* species.

Seq_1	Seq_2	Ka	Ks	Ka/Ks
GglaChr3G00053690.1	GglaChr6G00256870.1	0.173097	0.846342	0.204524
GglaChr5G00174780.1	GglaChr6G00256870.1	0.360528	1.761147	0.204712
GglaChr3G00053690.1	GglaChr5G00174780.1	0.374786	1.926632	0.194529
GglaChr1G00112760.1	GglaChr6G00254480.1	0.485632	1.364527	0.355897
GglaChr6G00258130.1	GglaChr7G00203880.1	0.383883	1.786748	0.21485
GglaChr1G00098810.1	GglaChr8G00283920.1	0.342888	0.727154	0.471548
GglaChr6G00231330.1	GglaChr7G00208870.1	0.266062	1.107452	0.240247
GglaChr4G00132430.1	GglaChr7G00208870.1	0.404922	1.545939	0.261926
GglaChr7G00208870.1	GglaChr7G00210960.1	0.371924	1.419957	0.261926
GglaChr4G00132430.1	GglaChr7G00210960.1	0.178359	0.581957	0.306482
GglaChr4G00140800.1	GglaChr7G00224680.1	0.177477	0.852525	0.208179
GglaChr1G00097870.1	GglaChr6G00257560.1	0.57205	1.812159	0.315673
GglaChr3G00052960.1	GglaChr6G00257560.1	0.347777	0.800537	0.43443
GglaChr6G00257560.1	GglaChr8G00285170.1	0.723128	1.99106	0.363187
GglaChr1G00097870.1	GglaChr8G00285170.1	0.332671	0.803466	0.414045
GglaChr1G00112760.1	GglaChr6G00248050.1	0.549365	1.626194	0.337822
Glycyrrhiza_uralensis_Fisch0006190.1	Glycyrrhiza_uralensis_Fisch0213770.1	0.519522	2.25986	0.229891
Glycyrrhiza_uralensis_Fisch0006190.1	Glycyrrhiza_uralensis_Fisch0207210.1	0.40337	2.802221	0.143947
Glycyrrhiza_uralensis_Fisch0174520.1	Glycyrrhiza_uralensis_Fisch0204820.1	0.501657	2.683818	0.186919
Glycyrrhiza_uralensis_Fisch0203500.1	Glycyrrhiza_uralensis_Fisch0241480.1	0.370956	1.79531	0.206625
Glycyrrhiza_uralensis_Fisch0020000.1	Glycyrrhiza_uralensis_Fisch0272010.1	0.252496	0.739061	0.341644
Glycyrrhiza_uralensis_Fisch0231350.1	Glycyrrhiza_uralensis_Fisch0246540.1	0.240374	0.950599	0.252865
Glycyrrhiza_uralensis_Fisch0132630.1	Glycyrrhiza_uralensis_Fisch0246540.1	0.371628	1.585172	0.23444
Glycyrrhiza_uralensis_Fisch0246540.1	Glycyrrhiza_uralensis_Fisch0248710.1	0.36665	1.004969	0.364837
Glycyrrhiza_uralensis_Fisch0132630.1	Glycyrrhiza_uralensis_Fisch0248710.1	0.184169	0.713102	0.258265
Glycyrrhiza_uralensis_Fisch0140980.1	Glycyrrhiza_uralensis_Fisch0262340.1	0.168614	0.800574	0.210616
Glycyrrhiza_uralensis_Fisch0021020.1	Glycyrrhiza_uralensis_Fisch0204110.1	0.495999	1.620524	0.306073
Glycyrrhiza_uralensis_Fisch0095640.1	Glycyrrhiza_uralensis_Fisch0204110.1	0.342205	0.807847	0.4236
Glycyrrhiza_uralensis_Fisch0021020.1	Glycyrrhiza_uralensis_Fisch0270930.1	0.304434	0.753878	0.403824
GuraChr2G074380.1	GuraChr4G131530.1	0.401037	2.646492	0.151535

GuraChr2G074380.1	GuraChr4G138160.1	0.39051	1.816979	0.214923
GuraChr4G129190.1	GuraChr5G178940.1	0.169708	0.89058	0.190559
GuraChr4G129190.1	GuraChr6G208380.1	0.364704	2.092954	0.174253
GuraChr5G178940.1	GuraChr6G208380.1	0.375007	2.002387	0.18728
GuraChr4G127810.1	GuraChr7G234850.1	0.412119	1.340285	0.307486
GuraChr2G060570.1	GuraChr8G275950.1	0.311855	0.793955	0.392786
GuraChr4G155230.1	GuraChr7G239740.1	0.242218	0.950599	0.254806
GuraChr1G027040.1	GuraChr7G239740.1	0.385884	6.210153	0.062138
GuraChr7G239740.1	GuraChr7G241940.1	0.364442	1.48847	0.244843
GuraChr1G027040.1	GuraChr7G241940.1	0.177425	0.602453	0.294505
GuraChr1G018710.1	GuraChr7G255680.1	0.168012	0.804861	0.208746
GuraChr2G059640.1	GuraChr4G128450.1	0.566923	1.993126	0.284439
GuraChr4G128450.1	GuraChr5G179700.1	0.329236	0.770054	0.427549
GuraChr4G128450.1	GuraChr8G277050.1	0.65782	1.482559	0.443706
GuraChr2G059640.1	GuraChr8G277050.1	0.325947	0.821597	0.396723
